# Diversity of Parallel Guanine Quadruplexes Induced by Guanine Substitutions

**DOI:** 10.3390/ijms21176123

**Published:** 2020-08-25

**Authors:** Klára Bednářová, Michaela Vorlíčková, Daniel Renčiuk

**Affiliations:** Institute of Biophysics of the Czech Academy of Sciences, Královopolská 135, 612 65 Brno, Czech Republic; bednarova@ibp.cz (K.B.); mifi@ibp.cz (M.V.)

**Keywords:** parallel guanine quadruplex, circular dichroism, stopped-flow, multimerisation, DNA secondary structure

## Abstract

Recently, we reported an inhibitory effect of guanine substitutions on the conformational switch from antiparallel to parallel quadruplexes (G4) induced by dehydrating agents. As a possible cause, we proposed a difference in the sensitivity of parallel and antiparallel quadruplexes to the guanine substitutions in the resulting thermodynamic stability. Reports on the influence of guanine substitutions on the biophysical properties of intramolecular parallel quadruplexes are rare. Moreover, such reports are often complicated by the multimerisation tendencies of parallel quadruplexes. To address this incomplete knowledge, we employed circular dichroism spectroscopy (CD), both as stopped-flow-assisted fast kinetics measurements and end-point measurements, accompanied by thermodynamic analyses, based on UV absorption melting profiles, and electrophoretic methods. We showed that parallel quadruplexes are significantly more sensitive towards guanine substitutions than antiparallel ones. Furthermore, guanine-substituted variants, which in principle might correspond to native genomic sequences, distinctly differ in their biophysical properties, indicating that the four guanines in each tetrad of parallel quadruplexes are not equal. In addition, we were able to distinguish by CD an intramolecular G4 from intermolecular ones resulting from multimerisation mediated by terminal tetrad association, but not from intermolecular G4s formed due to inter-strand Hoogsteen hydrogen bond formation. In conclusion, our study indicates significant variability in parallel quadruplex structures, otherwise disregarded without detailed experimental analysis.

## 1. Introduction

Guanine quadruplexes are secondary structures of nucleic acids, composed of several stacked square-shaped tetrads, each formed from four Hoogsteen hydrogen-bonded guanines [1]. This structure is stabilized by a cation, especially potassium, located in the central space between each pair of tetrads, and is also strengthened by crowding conditions [2,3]. These conditions facilitate the potential quadruplex occurrence in vivo. Potential quadruplex forming sequences (PQS) were found in genomes of numerous species [4] and, consequently, the guanine quadruplexes were indirectly observed both in purified genomic DNA [5] and in cells [6]. Based on the particular sequence, various types of quadruplexes have been described, differing in detailed structure, as well as in biophysical and biochemical properties [7,8]. Nucleic acids in cells are susceptible to various types of damage, some of them leading to base alterations and, subsequently, to altered nucleic acid secondary structure and binding properties. In the case of quadruplex-forming sequences, the influence of various base lesions has been thoroughly studied, but almost exclusively only on either conformationally highly variable *Vertebrate* telomere sequences (TTAGGG)_n_ [9,10] or model tetramolecular parallel quadruplexes TG_n_T [11]. In the case of intramolecular parallel quadruplexes, which represent a significant number of quadruplexes in the genome, only partial pieces of evidence have been reported [12,13]. Interestingly, the most stable types of intramolecular parallel quadruplexes seem to be depleted in genomes across species [14]. The absence of key guanine in PQS, in the reported case the substitution of central G of the first block by thymine, might be compensated by the coordination of water molecules in place of the missing guanine [15]; the quadruplex is formed, though it is significantly destabilized. In principle, the effect of mutations in PQS in genomic context might be also reduced by replacing either only the affected guanine by another guanine located outside the quadruplex-forming motif [16,17], or by replacing the whole affected G-tract with another [18,19,20]. For promoter quadruplexes such G-tract redundancy is quite common [21]. In both cases, the structure, biophysical properties and, consequently, the potential quadruplex interactome might be significantly affected. Studies of parallel guanine quadruplexes are complicated by the relatively narrow range of conditions suitable for their biophysical characterizations at physiological potassium concentration i.e., two-tetrad structures are quite reluctant to form [22], whereas three-tetrad ones are extremely stable [23]. Moreover, parallel quadruplexes tend to form multimers [24] even in cases where the primary sequence allows the formation of a monomolecular quadruplex. The multimerisation might result from several different modes [24,25], including terminal tetrad stacking with a strong 5′-5′ end stacking preference [26], reported also for tetramolecular parallel quadruplexes [27]. No interstrand guanine bonding is present here. The stacking of terminal tetrads might be prevented by the addition of a 5′ terminal overhang on the DNA sequence as shown for the HIV-1 integrase inhibitor T30695 quadruplex [28,29] and for the *oct4* gene promoter quadruplex [30].

In our former work, focusing on an inhibitory effect of guanine substitutions on the dehydration-induced conformation switch to parallel quadruplexes [31], we suggested a different sensitivity of parallel and antiparallel quadruplexes towards guanine substitution as one of the mechanisms behind the inhibitory phenomenon. We were thus interested how the lesions in guanines, simulated here by G to T substitutions, affect the properties of the parallel intramolecular quadruplexes and to which extent the guanines within each tetrad differ in terms of the effect of their substitution on the quadruplex properties.

## 2. Results and Discussion

**Figure 1:**
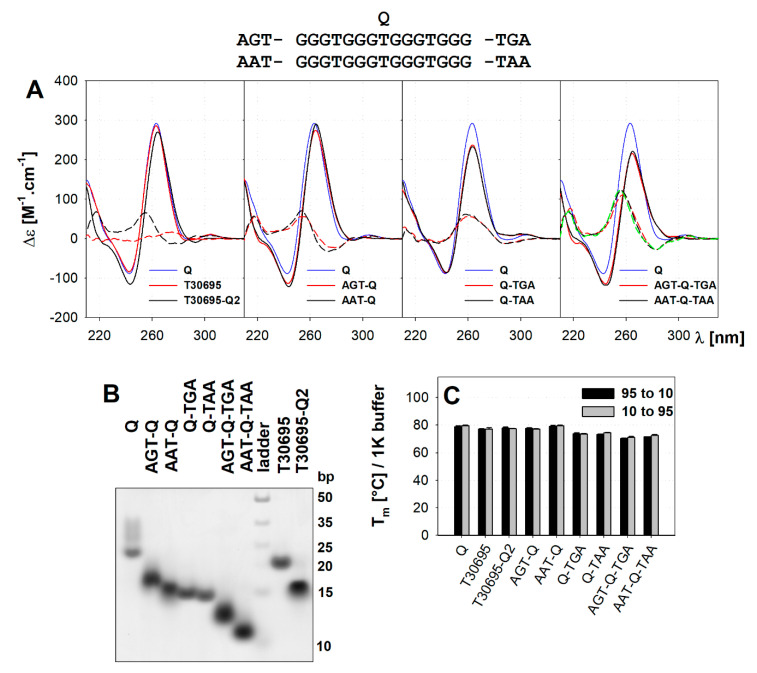
Model parallel quadruplexes with/without terminal overhangs: (**A**) CD spectra measured in 100K buffer at 20 °C; solid curves represent CD spectra, dashed curves represent difference to the Q spectrum; green line represents sum of differences of left and right overhang for AAT…TAA variant. (**B**) Native PAGE performed in 100K buffer at 20 °C. (**C**) Melting temperatures measured in 1K buffer, pH 7.

We commenced the study with the simplest model of potential intramolecular three-tetrad parallel quadruplexes, (G_3_T)_3_G_3_ (Q; 15 nt long) (Table S1; Figure 1), sequentially very close to the reported antiproliferative quadruplex T30695 that has only one 3′ additional thymine [32]. We followed its properties in 100K (10 mM potassium phosphate buffer, pH 7 with 85 mM KCl) and 1K buffer (1 mM potassium phosphate buffer, pH 7).

### 2.1. The Multimerisation of Parallel Quadruplexes

The T30695 quadruplex, or Q-T in our terminology, was shown to form a 5′-5′ end stacked dimer and this dimerization might be prevented by the addition of a two-thymine 5′ overhang, resulting in the sequence T30695-Q2 (TT-Q-T) [28]. For the following studies, we had to first exclude the quadruplex multimerisation to better reflect the natural biological localization of G4 and to simplify the determination of the effects of particular guanine substitutions in our model system. Q migrates on native PAGE as several smeared bands (Figure 1B) ranging from ~20 bp up to ~35 bp, i.e., even the fastest band is significantly slower than the bimolecular species. Any addition of terminal overhangs leads to an increase in PAGE migration speed, but the migration speed cannot be simply attributed to the molecularity of the complex. There is still a significant difference in migration between Q and T30695 suggesting that the Q might form even higher than bimolecular species, such as tetramolecular etc, although the 5′-5′ (head-to-head) end stacking seems to be preferred over 3′-3′ (tail-to-tail) or 5′-3′ (head-to-tail) [25,33]. In the case of 5′-3′ stacking or the simultaneous presence of 5′-5′ and 3′-3′ end stacking even higher associates would be expected, but we did not observe any such species. The 3′ thymine of T30695, causing an increase in migration speed, compared to Q, probably does not act simply through the prevention of 3′-3′ end stacking and only minimal stacking over the 3′ surfaces was reported. T30695-Q2 with 5′-TT has a significantly higher PAGE migration speed and forms a monomer [28]. However, according to the duplex ladder, the size still corresponds rather to the bimolecular species then the intramolecular. We thus added 5′, 3′ or both terminal overhangs, namely 5′-AGT...TGA-3′ and 5′-AAT...TAA-3′, selected based on our former studies of the *oct4* gene promoter quadruplex [30]. All the single-overhang oligonucleotides (18 nt long) migrated around the 15 bp duplex marker and close to T30695-Q2 reported to form monomer, i.e., they migrated either as slowed down monomolecular species or as faster bimolecular species, only the AGT-Q is slightly slowed down (Figure 1B). Otherwise, there is no significant difference in migration between oligonucleotides with 5′ and with 3′ overhang, as well as between oligonucleotides with AAT/TAA and AGT/TGA overhangs. The oligonucleotides with both 5′ and 3′ overhang (21 nt long) migrate even faster i.e., as monomolecular species, although they are 3-nt longer. The simplest explanation reflecting the native PAGE data indicates that all single-overhang sequences form a dimer thus the ones with a 5′ overhang through 3′-end stacking and vice versa those with 3′ overhang through the 5′-end stacking. This presumes the formation of tetramer/dimer structures for T30695 and T30695-Q2, which is, however, in contrast to the reported dimer/monomer structures [28]. Considering the discrepancy between our data and previous results, we might also suggest a more complex migration behaviour involving a combined result of several factors: (i) The molecular weight corresponding to the oligonucleotide length and differs due to the overhangs. (ii) The molecularity, i.e., presence of multiple strands in the quadruplex, possibly a result of various modes of interaction, which do not lead to the same change in migration. We will further refer the hydrogen bonded interlocked G4 as bimolecular (second mode in [25]), compared to dimer, caused by 5′-5′ end stacking (first mode in [25]). (iii) The presence of terminal tetrads exposed to the solvent, which significantly slows down the migration. This is in line with reported difference in electrophoretic migration of parallel and antiparallel quadruplexes of similar molecular weight, as shown for example for the human telomere motif AG_3_(TTAG_3_)_3_ and its triple A-to-AP mutant [34]. Our suggestion is that Q migrates slowly due to the 5′-5′ dimerization that also covers the 5′ tetrads but leaves the 3′ ones exposed. The addition of 3′ overhangs to Q does not prevent 5′-5′ dimerization, but the 3′ tetrads are covered and such sequences migrate faster. 5′ overhangs prevent dimerization, but 3′ tetrads are exposed to the solvent and the final migration is comparable to the dimer of sequences with 3′ overhangs only. The presence of both overhangs prevents dimerization and covers both exposed terminal tetrads leading to a faster migration despite the biggest molecular weight of the DNA strands. The accelerating effect of covered tetrads counterbalance the effect of dimerization. The ratio of exposed terminal tetrads to the strands involved differs for monomolecular and bimolecular species thus we further use sequences with both terminal overhangs to prevent such variability, in addition to preventing dimerization.

All the above-mentioned oligonucleotides give molar CD typical for parallel quadruplexes with a dominant positive peak around 264 nm (CD264) in 100K buffer (Figure 1A). The CD264, however, differs between oligonucleotides ranging from 300 M^−1^.cm^−1^ for Q to 230 M^−1^.cm^−1^ for AGT-Q-TGA and AAT-Q-TAA. To highlight these variations we calculated the difference CD spectra (CD of Q minus CD of respective sequence; Figure 1A—dashed lines). Whereas the presence of a 3′ end overhang leads to a significant decrease in CD264, the 5′ overhang does not reduce the peak height but causes a small red shift by 1.2–2 nm and a significant change in the circular dichroism in the 210–240 nm region to more negative values. The CD spectra of sequences with both overhangs correspond to the sum of spectral changes caused by the 5′ and the 3′ overhang; this indicates that the effect of the two overhangs is independent. The spectral change does not depend on the overhang sequence, but on its position: different overhangs at the same end result in the same change of CD. The T30695 gives a CD spectrum almost identical to the Q, whereas the T30695-Q2 spectrum differs in almost the same way as spectra of other 5′ terminal extended sequences. The spectral changes associated with 3′ overhangs, i.e., decreased 264 nm peak compared to Q, also exist in 1K buffer (Figure S1), but those linked to 5′ overhang, i.e., the shift of the 264 nm peak and decrease of the 210–240 nm spectral region, do not exist in 1K buffer. There is a CD spectral difference between 1K and 100K buffer for sequences lacking any 5′ end overhang (Figure S2) and this difference is very similar to that between sequences with and without a 5′ overhang in 100K buffer. This might imply that the spectral difference follows from the 5′-5′ end stacking of quadruplexes. However, according to PAGE in 1K buffer (Figure S3), the T30695 without 5′ end overhang, but not the T30695-Q2, still migrates as a dimer in 1K buffer, though the migration might be influenced by the effects mentioned above. Q migrates as one clear band of dimer size, without any diffuse slower species, indicating that a low potassium ion concentration weakens the multimerisation tendency, similarly as reported previously [33]. The sequences with only a 5′ overhang migrate in 1K buffer faster (Figure S3) than sequences with only a 3′ overhang. This is opposite to the situation in 100K buffer (Figure 1B). These changes are not, however, big enough to clearly explain the changes in molecularity due to low potassium ion concentration.

We could not determine the T_m_ in 100K buffer due to the extreme stability of all these quadruplexes. We thus compared the stabilities in 1K buffer (Figure 1C and Figure S4), with respect to the observed differences in CD and electrophoretic migration between 1K and 100K buffer. Interestingly, we observed a significant destabilizing effect of 3′ terminal overhangs by ~5 °C (T_m_ ~73 °C) compared to the 78–79 °C of Q and sequences with 5‘ overhang only. This goes against the expected destabilization effect of 5′ overhangs that should prevent the formation of stabilizing 5′-5′ tetrad dimers. A recent computational chemistry report on two-tetrad parallel quadruplexes and their dimers indicate that terminal tetrad dimerization might improve the stability of the complex [22].The presence of both overhangs destabilizes quadruplexes for another 2–3 °C, i.e., for ~8 °C compared to Q. We did not observe any significant hysteresis between renaturation and denaturation melting profiles in 1K buffer, not even for sequences migrating as bimolecular species, including Q. Similarly, we did not observe any sign of possible dimer decomposition in pre-melting phases followed by CD in 100K buffer (Figure S5). This might indicate either dissociation of the dimer tightly connected to the melting of the quadruplex or an absence of significant change in CD connected to the dissociation of the dimer. The second, however, goes against the CD spectral differences between the 5′ and 3′ extended Q discussed above.

In conclusion, to ensure the absence of quadruplex dimerization, we suggest adding 3-nucleotide overhangs on both ends. For further studies, we choose the AAT-TAA overhangs.

### 2.2. The Mutated Parallel Variants Are Uniform in Some Aspects and Diverse in Others

**Figure 2:**
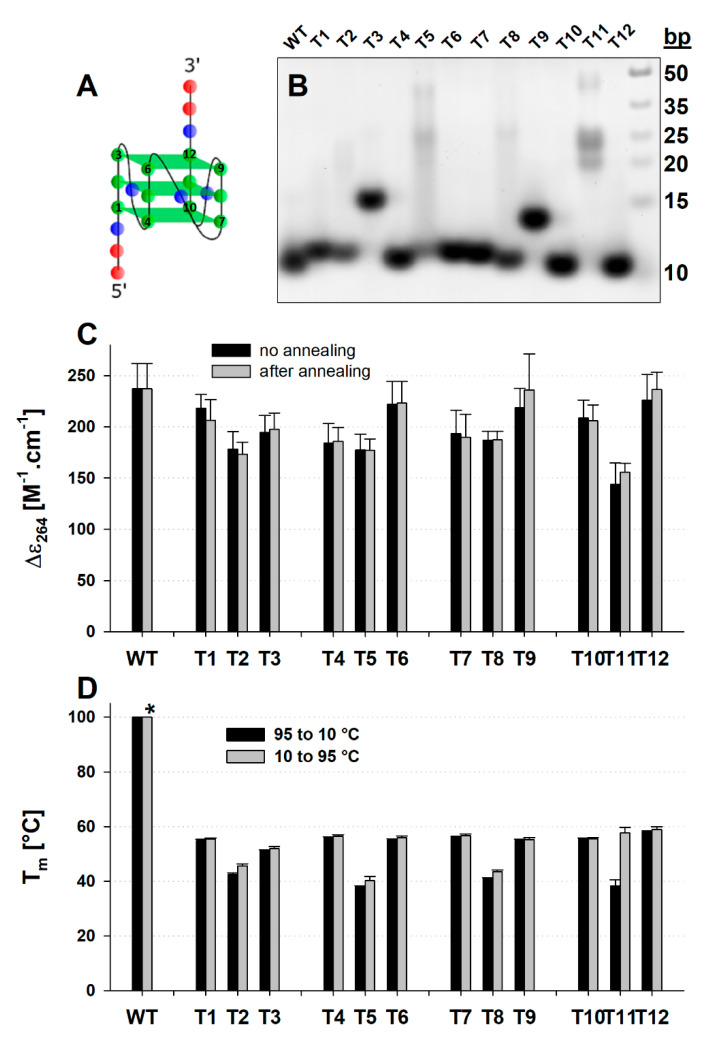
Different parameters of parallel WT sequence and all G/T mutants. (**A**) Schematic structure of the WT G4 with numbering of guanines used for labeling of G to T substituted variants. Green circles represent guanines, red ones adenines and blue circles represent thymines. Green trapezoids represent guanine tetrads. (**B**) Native PAGE. (**C**) The CD264 value (molar DNA strand circular dichroism (Δε) measured at 264 nm) before (black) and after (gray) thermal annealing. (**D**) T_m_ values observed from absorbance at 297 nm during renaturation (black) and denaturation (grey) processes. All data were observed in 100K buffer. Native PAGE and CD measurements were done at 23 °C. * T_m_ for WT could not be determined due to extreme thermal stability. Error bars in (**C**) and (**D**) represent standard deviations calculated from three independent measurements.

We then substituted individual guanines in AAT-Q-TAA (from now for simplicity labelled WT) with thymines to examine the similarity among guanines (Figure 2A). We followed the three parameters describing quadruplexes in the Section 2.1 in 100K buffer. (1) The thermodynamic stability represented by the T_m_ of both renaturation and denaturation processes (for simplicity incorrectly used also for T_1/2_ of multimolecular species). (2) The intensity of the CD signal at dominant positive peak around 264 nm (CD264). (3) The molecularity shown by the native PAGE migration of samples equilibrated in 100K buffer for about 30 min with or without thermal annealing. In addition, using a stopped-flow accessory we followed the kinetics of G4 formation, represented by changes of CD at 265 nm as a function of time, which should correspond to the folded fraction of the quadruplex. The wavelength 265 nm was selected due to the strong spectral line of the xenon-mercury lamp at 265 nm and close proximity to the CD264 used as a parallel quadruplex indicator. All mutated variants give CD spectra typical for parallel quadruplex (Figure S6) and the shape is principally the same for WT and all mutants. We thus followed only its amplitude (CD264) (Figure 2C). The intramolecular WT sequence gives a Δε_264_ of around 237 ± 25 M^−1^.cm^−1^, which is higher than the CD264 of any mutated variant. Interestingly, a high CD264 (218 to 226) was observed for T6, T9 and T12 variants of the 3′ terminal tetrad, but not for T3 in the same tetrad (CD264 ~ 194 ± 16 M^−1^.cm^−1^). Instead, T1 gives comparable values as 3′-tetrad variants. The T3, but to some extent also the T9, show significantly slower electrophoretic migration (Figure 2B), in the case of T3 between mono and bimolecular species. This is in contrast to the T1, T6 and T12 that are pure intramolecular species. The T_m_ decreases in order T12 (58 °C) > T6 and T9 (55 °C) to T3 (51 °C) (Figure 2D and Figure S7). As mentioned in Section 2.1, the T_m_ of WT in 100K buffer is above the range of possible determination by the method used. Compared to the WT, all G to T substituted variants are highly destabilized by at least 40 °C. All the four 5′ tetrad mutants (T1, T4, T7 and T10) migrate as pure intramolecular species (Figure 2B). They are almost equally thermostable (T_m_ 55 °C) with no significant hysteresis (Figure 2D and Figure S7). The CD264 ranges from T1 (218 ± 13 M^−1^.cm^−1^) to T4 (184 ± 19 M^−1^.cm^−1^) (Figure 2C). All four central tetrad mutants (T2, T5, T8 and T11) give a CD264 in a range between 186 M^−1^.cm^−1^ for T8 to 144 M^−1^.cm^−1^ for T11. The electrophoretic migration of these oligonucleotides ranges from pure intramolecular T2 over T8 with a small fraction of bimolecular species and T5 with an equal amount of intra- and multimolecular species, to T11, which is dominantly bimolecular in at least two different forms with a small fraction of tetramolecular species (Figure 2B). The decreased temperature (2 °C) of the electrophoresis run and the slow annealing of samples before the run led to a significant increase in the portion of multimolecular species for all mutant oligonucleotides (Figure S8), which is in line with recent observations on various parallel quadruplexes [33]. This tendency to form multimolecular species is reflected also by the hysteresis of T_m_ calculated from the denaturation and renaturation phases. This difference ranges from 3 °C for T2 and T8 to 15 °C for T11. The absolute T_m_ values calculated from the renaturation profile are in a range between 38 °C (T5) and 42 °C (T2) (Figure 2D and Figure S7). The denaturation profiles of T11 and, to a lesser extent of T2 and T8, show multi-phasic processes, whereas the melting curve of T5 follows quite well a mono-phasic transition. The presence of a multi-phasic melting profile and T_m_ hysteresis (Figure S7) correlates with the multimolecular behaviour of T11 (Figure 2B), but not of the T5. Interestingly, the T_m_ of T11 calculated from a more stable part of the denaturation phase, 55 °C, is close to that of the terminal tetrad mutants.

**Figure 3:**
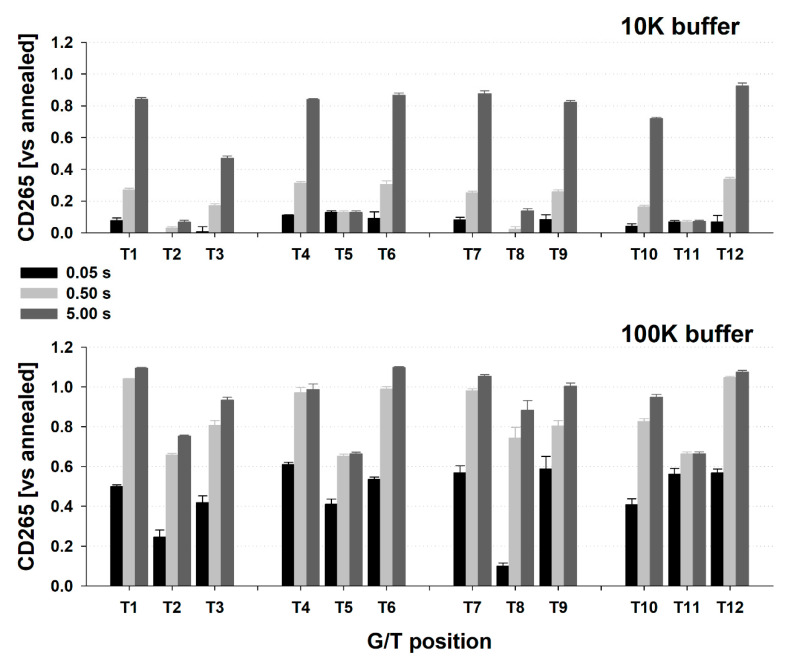
The relative portion of G4 folded calculated from circular dichroism for all G/T mutants in 10K (top) and 100K (bottom) buffer at various times after mixing. The values are based on Δε_265_, normalized to value observed by stopped-flow experiment mixing DNA with 1NF buffer (0) and to value measured after annealing in 100K buffer (1). Error bars represent standard deviations calculated from three independent measurements.

Using a stopped-flow accessory, we then followed the kinetics of quadruplex formation upon mixing the DNA in 1 mM sodium phosphate buffer, pH 7 (1NF buffer) with a 2x concentrated potassium buffer giving a final concentration of 10 mM K^+^ (10K buffer) or 100 mM K^+^ (100K buffer), respectively (see Section 3. Materials and Methods for precise buffer composition). We expressed the kinetics as a fraction of DNA folded into a quadruplex (Figure 3), calculated from the Δε value at 265 nm (Figure S9). The fully folded state (value 1) corresponds to the averaged Δε_265_ value observed from CD spectra measurements after thermal annealing during the melting experiments above in Section 2.2. This calculation allows the gaining of values above 1 in cases where there is a decrease in CD265 with annealing. The unfolded state (value 0) corresponds to the Δε_265_ value of DNA in 1NF buffer after mixing and is very close to the Δε_265_ in 1NF buffer at 90 °C. From the fit of the experimental data, we then calculated the fraction folded at 50 ms, 500 ms and 5 s. The WT sequence is partially folded into quadruplexes already in 1NF buffer after denaturation and fully folded in the dead time of the instrument in 1K buffer [35]. The extremely fast folding kinetics of WT might come from an incomplete thermal denaturation of the quadruplex and the presence of various pre-folded states [36]. In contrast, none of the G/T substituted variants form a significant portion of folded quadruplexes in 1K buffer within the 5 s of measurement [35].

In 10K buffer, all terminal mutants except T3 are about 75% folded to G4 within five seconds of the stopped-flow (SF) experiment and about 20–25% is folded already in 500 ms (Figure 3). The T3 folds much slower than the other terminal mutants with only about 45% folded after 5 s. In contrast, only a small fraction of central mutants is folded. In 100K buffer, at least 25% of each terminal mutant is folded within the 50 ms, with almost 60% of T9 folded (Figure 3). 75% (T3) to 90% (T1) is folded within 500 ms. Interestingly, the T5 and T11 central mutants fold partially (50–70%) into G4 within 500 ms, but further increase of Δε_265_ and achievement of 100% takes tens of minutes i.e., for the variants tending more to form multimolecular species, there is no change in Δε_265_ between 500 ms and 5 s. These experiments show an average over many molecules and over the data point acquisition time, thus we cannot distinguish whether the 50% Δε at 265 nm after 5 s in 100K corresponds to a partially folded intermediate, probably intramolecular due to the fast kinetics of folding, that is formed by all molecules in solution, or whether it reflects a folding-unfolding equilibrium with about 50% of fully folded molecules on average. As we followed only the changes of Δε at 265 nm, we cannot determine whether the formation of parallel quadruplexes of all mutated variants proceeds directly from the unfolded state to the parallel one, or through an antiparallel intermediate as was recently observed for a sequence quite similar to WT [37]. From end-point CD spectra, significant Δε around 290 nm corresponding to a fraction of antiparallel quadruplex or *syn* geometry of guanines were not observed [13] and there was no significant CD spectral difference between annealed and non-annealed samples.

Interestingly, although the absorbance at around 265 nm is not usually considered as a suitable indicator of G4 folding/unfolding and the region around 295 nm is a better choice [38,39], we observed a rather high linear correlation between the stopped-flow assisted record of CD and the absorbance at 265 nm [35]. There is, however, a difference between CD and absorbance in the relative start of the data record during the stopped-flow experiment, when normalized to a DNA-with-1NF buffer mixing experiment as a starting value even for records performed in 10K buffer (Figure 3 vs. Figure S10). The CD record (y0 parameter of the fitting equation) starts at values close to the level observed with 1NF, i.e., unfolded state, whereas absorbance at the start of the data record is already at about 40% of the final value of the fully folded G4. The potassium buffers used here do not cause such an increase in absorbance at 265 nm. It should be noted that the CD and absorbance records during stopped-flow experiment are taken simultaneously.

In 10K buffer the SF-absorbance record of G4 formation is very well fitted by the 3-parametric function, indicating a simple two-state process in the time scale of the experiment. In 100K, however, the residuals of the 3-parametric and 5-parametric fits indicate that there might exist two processes in five-second records during our experiments, which is in line with former reports [39]: a fast one taking place within hundreds of milliseconds and responsible for about 90% of the signal change, and a slow one taking place on a tens of seconds scale that is responsible for the remaining 10%. The two-process behaviour is significant for terminal tetrad mutants, whereas for central tetrad mutants only a single process, fitted by a simple 3-parametric function, is observed in the timescale. The difference in the calculated folded fraction between using 3-parametric and 5-parametric functions is within the standard deviation of the 3-parametric fit. The quality of the data does not allow to unambiguously follow similar single and double process behaviour by CD record.

As the last parameter compared, we followed the interaction of WT and its substituted variants with N-methyl mesoporphyrin IX (NMM) (Figure S11), a reported G4-specific ligand with strong preference towards parallel G4 type [40]. NMM preferentially interact with planar terminal tetrads of parallel quadruplex [41] and terminal tetrads surfaces might be potentially strongly selectively affected by G/T substitutions. Due to the high thermal stability of WT we were not able to compare the NMM stabilizing effect of WT. All G4 of G/T substituted variants are stabilized by 8–15 °C with T10 being the most stabilized one. Interestingly, we did not observe any tetrad preference for NMM-linked G4 stabilization. NMM interaction does not significantly affect the CD of the quadruplexes.

In summary, some of the G4s with single G to T substitution in the same tetrad differ in various properties. In principle, according to the literature, the quadruplexes formed by mutated variants might be to some extent rescued by the coordination of a water molecule at the position of the replaced guanine [15], while substituting thymine is flipped out of the core. Single G to T substitution and possible flipping out of the T also leads to the formation of a G-register [42], affecting the overall averaged properties of G4. Both the water substitution and the register formation susceptibility would probably differ depending on the position of the substitution.

### 2.3. The DNA Concentration-Dependent Formation of Type 2 Multimolecular Structures Is Not Directly Reflected in CD

**Figure 4:**
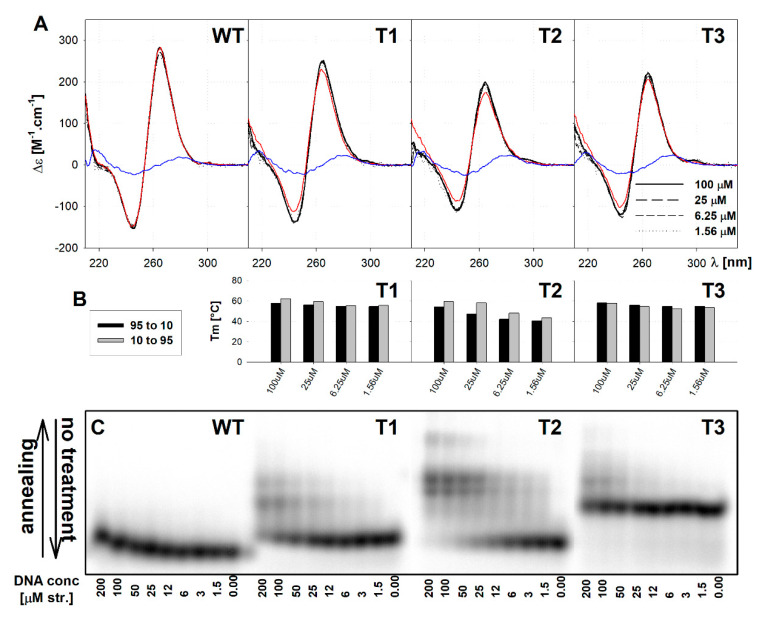
Dependence of the G4 properties of WT and selected mutated sequences on DNA concentration in 100K buffer. (**A**) The CD spectra (**B**) T_m_ values from renaturation (black) and denaturation (gray) profile and (**C**) native PAGE o. Red CD spectra correspond to samples slowly annealed in 100K buffer at 100 µM DNA strand concentration. Blue CD spectra correspond to denatured samples measured at 98 °C in 1Nf buffer at 3 µM strand concentration.

We continued our studies using the WT, T1, T2 and T3 sequences because they differ in the properties described in Section 2.2, mainly in electrophoretic migration behaviour, and examined them at various DNA concentrations between 200 µM and 0.01 µM DNA strand to follow their susceptibility to forming higher structures. The CD spectra (Figure 4A) of all tested oligonucleotides did not change from 100 µM to 1.5 µM DNA strand concentration. In contrast to the CD, the molecularity of the quadruplexes significantly differed for all mutated quadruplexes (Figure 4C); the WT sequence remained intramolecular within the whole DNA concentration range tested, though we observed a small slow-down of migration with increasing DNA concentration. The T1 continuously transformed to at least two equally populated bimolecular species with intra-to-bi transition midpoint at around 100 µM strand concentration. The T2, dominantly intramolecular around 3 µM DNA, transformed around 20 µM DNA to at least two bimolecular species, similarly as T1, with a significant presence of tetramolecular species at the expense of monomolecular ones above 100 µM DNA. The T3, originally migrating as a faster bimolecular species retained this speed over the whole tested concentration range, only at above 100 µM DNA a small population of more slowly migrating species appeared. For all variants, there is an increase in Tm (T1/2) with increasing DNA concentration (Figure 4B and Figure S12) and this effect is most pronounced for T2, which reaches the Tm of T1 or T3 at 100 μM DNA. T2 and, to a lesser extent also T1, shows hysteresis between Tm calculated from the renaturation and denaturation processes, reflecting the increasing amount of multimolecular species seen on PAGE. We observed only minimal hysteresis in Tm for T3. The concentration-dependent experiments indicate that the T3 is a monomolecular structure with an altered structure resulting in an anomalous migration rather than a dimer or bimolecular G4.

### 2.4. Parallel Quadruplexes Are Significantly More Affected by Guanine Lesions than the Antiparallel Ones

**Figure 5:**
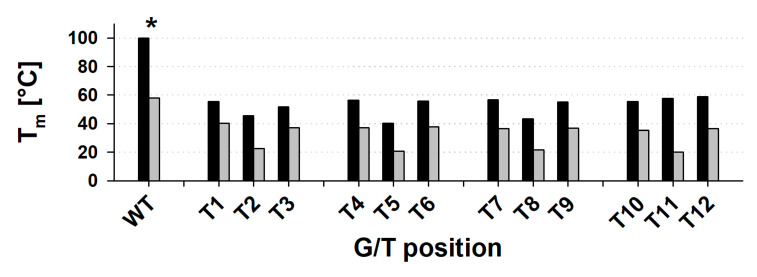
The T_m_ of parallel (black) and antiparallel (gray) quadruplexes for WT sequences and all G/T substituted variants in 100K buffer. *—The T_m_ of parallel WT sequence could not be determined because of the extreme stability of the parallel WT quadruplex in 100K buffer.

Finally, we were interested whether there is a difference in the response of parallel and antiparallel quadruplexes to the guanine substitution as a possible explanation of an inhibitory effect of guanine substitutions on the dehydration-induced shift of antiparallel to parallel quadruplexes [31]. The effect of various guanine substitutions on different non-parallel guanine quadruplexes has previously been reported [9,10]. For a brief comparison, we measured variants of AAT(G_3_T_3_)_3_G_3_TAA sequence (aWT; Table S1) with individual guanines replaced with thymines. aWT sequence, as well as all variants with terminal substitutions form, according to the CD spectra (Figure S13) similar, but non-parallel G4 types. Precise G4 conformation cannot be determined from these CD; they resemble to some extent variants of 22-mer of human telomere sequence in potassium, whose conformation is still a matter of debate [34]. Variants of aWT with substituted guanines 2, 5, 8 and 11, i.e., potentially located in central tetrad of a three-tetrad G4, give slightly different CD spectra due to presence of unfolded species. This is reflected in T_m_, which ranges between 57 °C for aWT to around 20 °C of central tetrad-substituted variants (Figure S14). For all substituted variants, the destabilizing effect of G to T substitution is much higher in parallel G4 than in antiparallel (Figure 5). The difference in stability of WT sequences, exceeds 50 °C; the parallel quadruplex T_m_ is higher than 100 °C and cannot be determined, whereas the WT antiparallel quadruplex T_m_ is only 57 °C. Unlike the absolute T_m_ values, there is only mild position dependence of the difference in T_m_ between parallel and antiparallel variants and there is no projection of the central G mutations as they are less stable in both parallel and antiparallel form. From the perspective of the previous work [31], the hypothesis that the inability of a conformational switch from antiparallel to parallel G4 after guanine substitution is caused by a substantial relative decrease of the thermal stability of the potential target parallel structure, compared to the initial antiparallel structure, could well be true. Although the results reported here show a still higher thermal stability of parallel forms, the change in loop length that significantly influences the thermal stability [43,44], is not taken into account.

## 3. Materials and Methods

Synthetic oligonucleotides were purchased from Sigma-Aldrich (Haverhill, UK). All oligonucleotides and sequences used or discussed in this work are DNA. For ease of understanding we did not follow the strict d(...) notation. Oligonucleotides were desalted and lyophilized by the provider. Lyophilized oligonucleotides were dissolved in 1 mM sodium phosphate buffer, pH 7 with 0.3 mM EDTA (1NF buffer). The precise concentration of the oligonucleotides for measurements was determined from UV absorption at 260 nm in the buffer at 90 °C using molar absorption coefficients calculated according to Gray [45]. The purity of the oligonucleotides was checked by denaturing polyacrylamide gel electrophoresis (PAGE). All sequences and their labels used are summarized in Table S1. Unless stated otherwise, all measurement experiments were conducted either in 1 mM potassium phosphate buffer, pH 7 (1K buffer) or in 10 mM potassium phosphate buffer at pH 7 with 85 mM KCl (100K buffer) at around a 70 µM DNA nucleoside concentration at 20 °C. For stopped-flow experiments (Section 2.2 and Figure 3), we used also the 10K buffer, which represents 100K buffer mentioned above diluted 10-times by 1 mM sodium phosphate buffer, pH 7. In Section 2.1 and Section 2.2, the spectroscopic and thermodynamic data are calculated as an average of three independent experiments with standard deviation values, where applicable.

Circular dichroism (CD) measurements were conducted on a Jasco J-815 dichrograph (Jasco Corp.,Tokyo, Japan). Particular CD spectra were collected as an average of four measurements between 330 nm and 210 nm with data pitch 0.5 nm at 200 nm.min^−1^ acquisition speed. CD signals are expressed as the difference in the molar absorption (Δε) of left- and right-handed circularly polarized light, and the molarity is related to strands. The experimental conditions were changed directly in the cells by adding stock solutions of respective salts. The final concentration was corrected for the volume increase. DNA before measurement was annealed in these conditions by heating to 90 °C for 5 min, followed by cooling down to room temperature over several hours. The spectra in Section 2.1 and Section 2.2 are an average of three independent experiments. In these two sections and contrary to the rest of CD spectra, the DNA concentrations used for conversion to molar units were calculated from absorption at 260 nm from spectra taken between denaturation and renaturation phase of UV melting experiment, i.e., at maximal temperature (95 °C) already in respective buffer. It has to be noted that the melting experiments were performed with identical DNA solutions as the CD measurements in Section 2.1 and Section 2.2. Samples in Section 2.1, measured in 100K, were prepared from samples in 1K by addition of respective salts and the concentration was calculated from that of the 1K conditions using the correction for volume increase. This approach was used due to extreme stability of these samples in 100K buffer and inability to obtain absorption spectra of denatured samples. The effect of salt concentration on the absorption at 260 nm was negligible.

Stopped-flow assisted kinetic measurements of G4 formation were performed using a Chirascan Plus a dichrograph equipped with a Stopped-Flow accessory (Applied Photophysics, Leatherhead, UK) and a xenon-mercury lamp. The device was set to 265 nm, 1 nm bandwidth and 1 cm optical path length and both circular dichroism and absorbance data were collected. DNA concentration was precisely set to 7.8 µM strand. Five 1:1 mixing reactions, each with eight repeats, were consecutively measured for each oligonucleotide: 1NF buffer with 1NF buffer, DNA with 1NF buffer, DNA with 100×, 10× and 1× diluted 20 mM potassium phosphate buffer, pH 7 with 170 mM KCl (2× concentrated 100K buffer). After 1:1 mixing, the DNA was in 100K buffer for 1× dilution. Each repeat consisted of 5000 experimental points, 1 ms each, i.e., 5-s kinetics were obtained. At least five selected experimental traces (repeats) of each reaction and oligonucleotide were baseline-corrected i.e., an average of 1NF with 1NF buffer reaction was subtracted, then recalculated into Δε or ε units and, finally, fitted by a single-exponential rise to maximum function y = y0 + a*(1 − e^(−b*x)^) using a least squares sum and gradient method in Excel (Microsoft Corp., Redmond, WA, USA). After each reaction, a CD spectrum of the mixed solution was collected (210 to 330 nm, 0.5 nm step, 0.5 s data integration time).

Ultraviolet (UV) absorption-based melting experiments were conducted on a Varian Cary 4000 UV/Vis spectrophotometer (Varian, Mulgrave, Australia). Whole spectra were taken within two temperature ramps ranging from 95 °C to 10 °C and 10 °C to 95 °C, respectively, in 1 °C steps. The average temperature decrease/increase rate was approximately 0.25 °C.min^−1^. Each spectrum was measured between 330 nm and 230 nm with data pitch 1 nm at a scan rate of 600 nm.min^−1^. In the figures, the melting curves are expressed as the change in molar absorption (ε) at 297 nm of DNA strands i.e., the two baseline corrected melting curves [46]. T_m_ values were calculated from such normalized curves as the temperature where half of the sample is folded. The presented T_m_ values, as well as the corresponding melting curves, are an average of three independent experiments.

Non-denaturing polyacrylamide gel electrophoresis (PAGE) was performed in a thermostated SE-600 instrument (Hoefer Scientific, San Francisco, CA, USA). Gels (16% PA, 29:1 mono/bis ratio, 14 × 16 × 0.1 cm in size) containing the appropriate salt concentration were run at 40 V and 20 °C for 16 h. Either 2 µg of DNA in 20 µl of buffer (Stains-all stained gels) or various amounts of DNA (fixed amount of ^32^P labelled DNA supplemented with a precise concentration of non-labelled DNA) were loaded into each lane. 5′-labeled samples were prepared using ^32^P-γ-ATP and T4-polynucleotide-kinase. Before loading on the gel, DNA was thermally denatured in 1 mM sodium phosphate buffer, pH 7 with 0.3 mM EDTA, and the buffer and salt concentration were adjusted. The gels were either stained with Stains-all dye (Merck / Sigma-Aldrich, St. Louis, MO, USA) and digitalized using Personal Densitometer SI 375-A (Molecular Dynamics, Sunnyvale, CA, USA) or the gels with ^32^P labelled DNA (see Section 2.3) were exposed to a PhosphoImager screen for 2h and scanned using a Typhoon FLA9000 device (GE Healthcare, Chicago, IL, USA).

## 4. Conclusions

The work presented here follows our former reports on the effect of various guanine substitutions on the structure and stability of guanine quadruplexes. Special emphasis was given to identify the reason for the inability of imperfect quadruplex-forming sequences to undergo a conformational transition from an antiparallel/hybrid type to a parallel one due to crowding/dehydrating agents that might partially simulate crowded in-cell conditions. We observed that parallel quadruplexes are highly sensitive towards guanine substitutions, compared to antiparallel ones, which might influence the final structural equilibrium of the dehydration-induced transition. Moreover, we have shown that guanine substitutions at specific positions within one tetrad are not fully equal in terms of their effect on stability, structure and the tendency to form multimers. We also observed indices that different types of multimerisation might be distinguished by circular dichroism spectroscopy. In conclusion, these data will serve as an informative background for ongoing studies of G4 repertoire in more biology-oriented systems

## Supplementary Materials

Supplementary materials can be found at https://www.mdpi.com/1422-0067/21/17/6123/s1. Figure S1: Averaged CD spectra from three experiments of model parallel quadruplexes with various terminal overhangs measured in 1K buffer at 20 °C; solid curves represent CD spectra, dashed curves represent difference to the spectrum of Q. Figure S2: Averaged CD spectra from three experiments of model parallel quadruplexes with various terminal overhangs measured in 1K buffer (blue) and in 100K buffer (red). The difference between the two spectra is in black. Figure S3: Native PAGE performed in 1K buffer (1 mM potassium phosphate buffer, pH 7) at 20 °C. Oligonucleotides were prepared in the same buffer. Figure S4: Averaged UV melting curves from three experiments of model parallel quadruplexes with various terminal overhangs measured in 1K buffer and expressed as folded fraction (0-1 normalized curves) of G4 during renaturation (black) and denaturation (red). Figure S5: Left: CD spectra of Q (A, B) and AAT-Q-TAA (D, E) in 1K (A, D) and 100K (B, E) buffer at selected temperatures between 20 and 85 °C. Right: Δε at 264 nm (black) and 220 nm (red) of Q (C) and AAT-Q-TAA (F) as a function of temperature, measured in 1K (solid circles) and in 100K buffer (empty circles). Figure S6: Averaged CD spectra from three experiments of WT (red) and G/T mutated variants (black) of model parallel quadruplex measured in 100K buffer at 23 °C. Figure S7: Averaged UV melting curves from three experiments of G/T mutated variants measured in 100K buffer and expressed as folded fraction (0-1 normalized curves) of G4 during renaturation (black) and denaturation (red). WT is not shown due to extreme thermal stability. Figure S8: Native PAGE performed in 100K buffer at 20 °C (upper panel) or at 2 °C (bottom panel). Oligonucleotides were annealed in 100K buffer over 3 h from 90 °C to 20 °C or 2 °C. Figure S9: Left panels: Δε at 265nm measured each millisecond for 5s total after mixing sample with 2 mM K^+^ (blue), 20 mM K^+^ (red) or 200 mM K^+^ (green) using a stopped-flow accessory. Black lines represent a three-parameter exponential fits (“rise-to-maximum”) of the experimental points. Right panels: CD spectra of respective samples measured right after particular stopped-flow experiments. Black solid spectrum represents average of three independent measurements of sample prepared in 100K buffer by standard procedure. Figure S10: The relative portion of G4 folded calculated from absorbance for all G/T mutants in 10K (top) and 100K (bottom) buffer at various times after mixing. The values are based on ε_265_, normalized to value observed by stopped-flow experiment mixing DNA with 1NF buffer (0) and to value measured after annealing in 100K buffer (100). Figure S11: (A) The CD264 value (molar DNA strand circular dichroism (Δε) measured at 264 nm) of G to T substituted variants measured in 100K in absence (black) or presence (gray) of two equivalents of NMM. (B) Difference in T_m_ values calculated from samples with and without two equivalents of NMM from renaturation (black) or denaturation (gray) profile measured in 100K buffer. T_m_ of WT is not shown due to extreme thermal stability. Figure S12: UV melting curves of G/T mutated variants measured in 100K buffer at various concentration and expressed as folded fraction (0-1 normalized curves) of G4 during renaturation (black) and denaturation (red). WT is not shown due to extreme thermal stability. Figure S13: CD spectra of aWT (red) and G/T mutated variants (black) of model hybrid/antiparallel quadruplex measured in 100K buffer at 23 °C. Figure S14. UV melting curves of aWT and G/T mutated variants measured in 100K buffer and expressed as folded fraction (0-1 normalized curves) of G4 during renaturation (black) and denaturation (red). Table S1: Oligonucleotides used in the study.

## Abbreviations

G4	Guanine quadruplex
CD	Circular dichroism
PAGE	Polyacrylamide gel electrophoresis
SF	Stopped-flow
