# Supplementary material for: Diversity of Parallel Guanine Quadruplexes Induced by Guanine Substitutions
Source: Int J Mol Sci. 2020 Aug 25;21(17):6123. doi: 10.3390/ijms21176123 (PMC7503932; doi:10.3390/ijms21176123)

# SUPPLEMENTARY INFORMATIONS

**Table S1.** Oligonucleotides used in the study.

| Label | Sequence |
|---|---|
| Q | GGGTGGGTGGGTGGG |
| T30695 | GGGTGGGTGGGTGGGT |
| T30695TT | TTGGGTGGGTGGGTGGGT |
| Q-TGA | GGGTGGGTGGGTGGGTGA |
| Q-TAA | GGGTGGGTGGGTGGGTAA |
| AGT-Q | AGTGGGTGGGTGGGTGGG |
| AAT-Q | AATGGGTGGGTGGGTGGG |
| AGT-Q-TGA | AGTGGGTGGGTGGGTGGGTGA |
| AAT-Q-TAA = WT | AATGGGTGGGTGGGTGGGTAA |
| T1 | AATTGGTGGGTGGGTGGGTAA |
| T2 | AATGTGTGGGTGGGTGGGTAA |
| T3 | AATGGTTGGGTGGGTGGGTAA |
| T4 | AATGGGTTGGTGGGTGGGTAA |
| T5 | AATGGGTGTGTGGGTGGGTAA |
| T6 | AATGGGTGGTTGGGTGGGTAA |
| T7 | AATGGGTGGGTTGGTGGGTAA |
| T8 | AATGGGTGGGTGTGTGGGTAA |
| T9 | AATGGGTGGGTGGTTGGGTAA |
| T10 | AATGGGTGGGTGGGTTGGTAA |
| T11 | AATGGGTGGGTGGGTGTGTAA |
| T12 | AATGGGTGGGTGGGTGGTTAA |
| aWT | AATGGGTTTGGGTTTGGGTTTGGGTAA |
| aT1 | AATTGGTTTGGGTTTGGGTTTGGGTAA |
| aT2 | AATGTGTTTGGGTTTGGGTTTGGGTAA |
| aT3 | AATGGTTTTGGGTTTGGGTTTGGGTAA |
| aT4 | AATGGGTTTGGTTTGGGTTTGGGTAA |
| aT5 | AATGGGTTTGTGTTTGGGTTTGGGTAA |
| aT6 | AATGGGTTTGGTTTTGGGTTTGGGTAA |
| aT7 | AATGGGTTTGGGTTTTGGTTTGGGTAA |
| aT8 | AATGGGTTTGGGTTTGTGTTTGGGTAA |
| aT9 | AATGGGTTTGGGTTTGGTTTTGGGTAA |
| aT10 | AATGGGTTTGGGTTTGGGTTTTGGTAA |
| aT11 | AATGGGTTTGGGTTTGGGTTTGTGTAA |
| aT12 | AATGGGTTTGGGTTTGGGTTTGGTTAA |

**Figure S1.** Averaged CD spectra from three experiments of model parallel quadruplexes with various terminal overhangs measured in 1K buffer at 20 °C; solid curves represent CD spectra, dashed curves represent difference to the spectrum of Q.

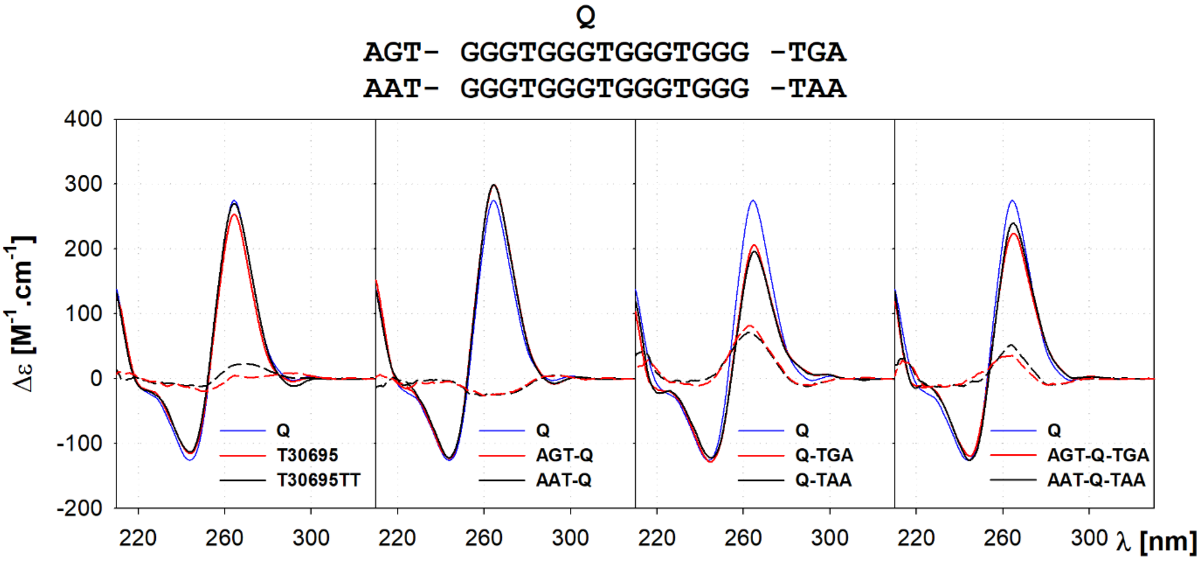

**Figure S2.** Averaged CD spectra from three experiments of model parallel quadruplexes with various terminal overhangs measured in 1K buffer (blue) and in 100K buffer (red). The difference between the two spectra is in black.

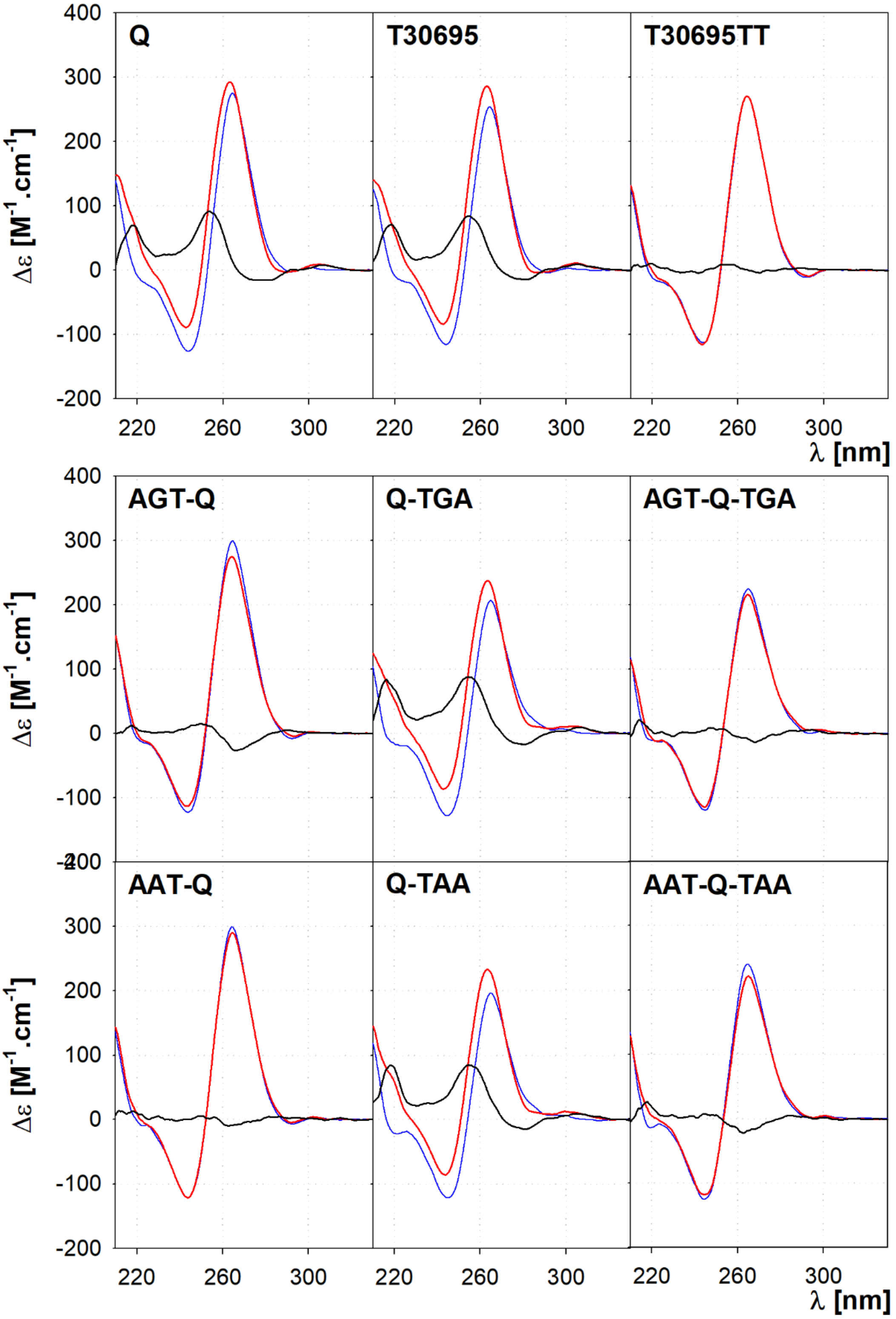

**Figure S3.** Native PAGE performed in 1K buffer (1 mM potassium phosphate buffer, pH 7) at 20 °C. Oligonucleotides were prepared in the same buffer.

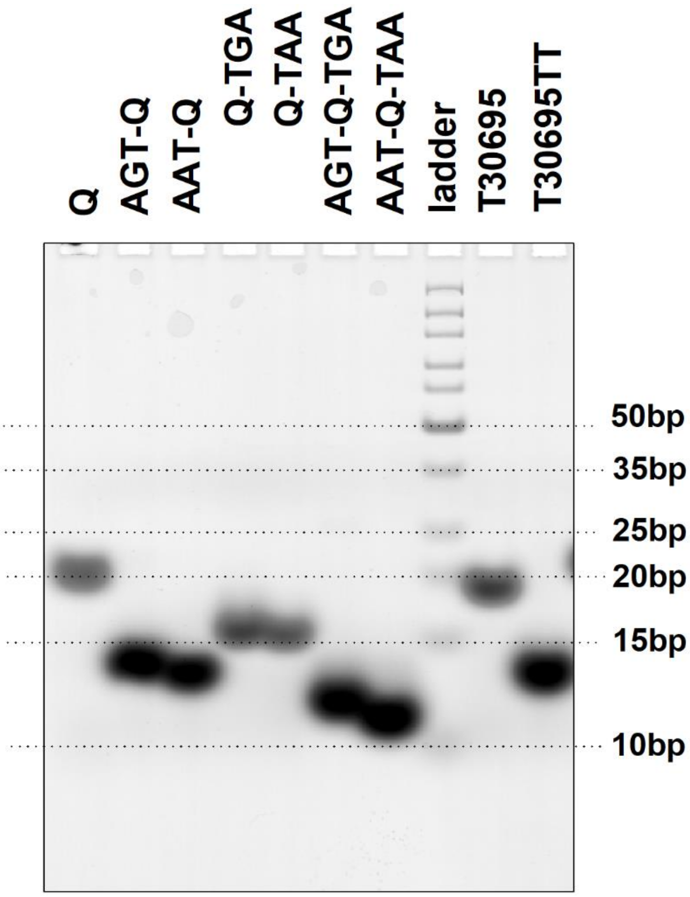

**Figure S4.** Averaged UV melting curves from three experiments of model parallel quadruplexes with various terminal overhangs measured in 1K buffer and expressed as folded fraction (0-1 normalized curves) of G4 during renaturation (black) and denaturation (red).

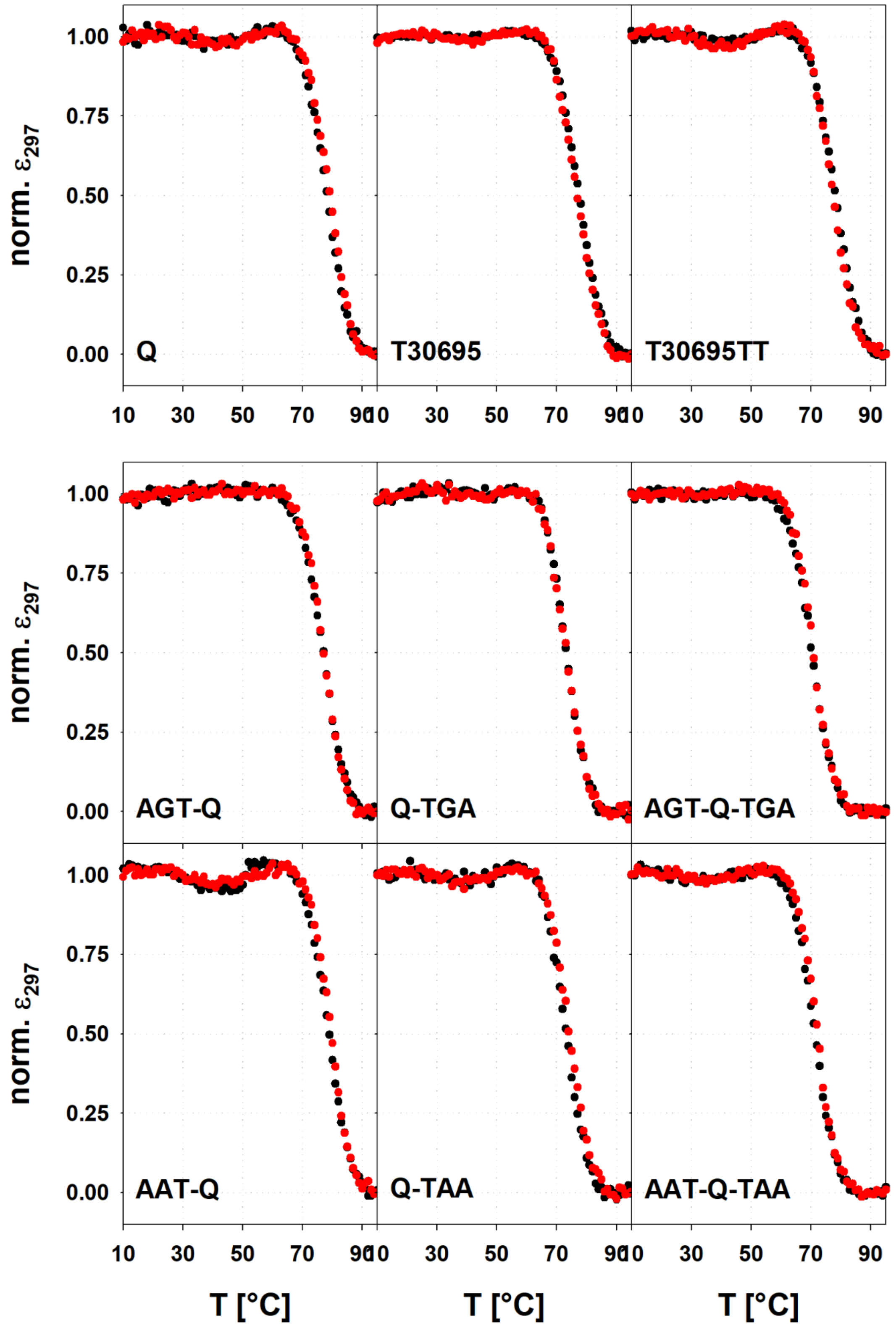

**Figure S5.** Left: CD spectra of Q (**A**,**B**) and AAT-Q-TAA (**D**,**E**) in 1K (**A**,**D**) and 100K (**B**,**E**) buffer at selected temperatures between 20 and 85 °C. Right: Δε at 264 nm (black) and 220 nm (red) of Q (**C**) and AAT-Q-TAA (**F**) as a function of temperature, measured in 1K (solid circles) and in 100K buffer (empty circles).

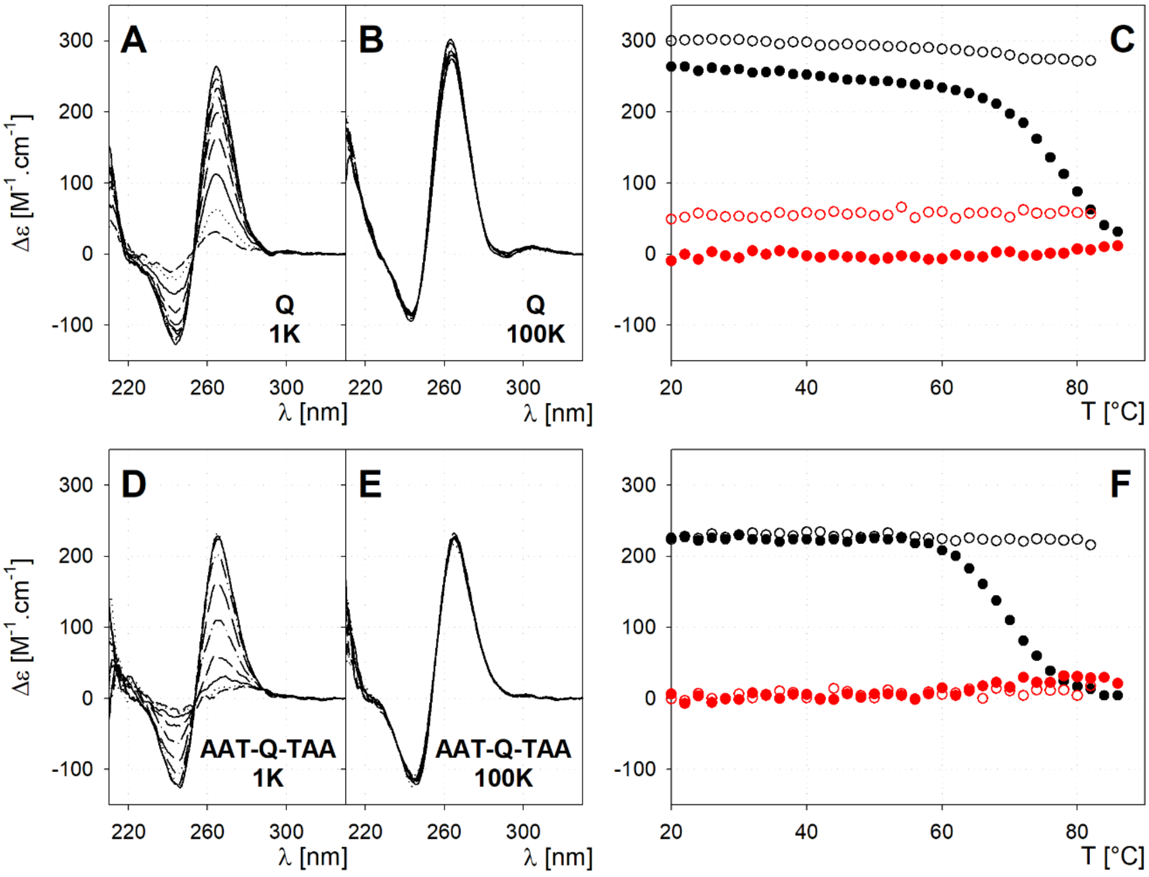

**Figure S6.** Averaged CD spectra from three experiments of WT (red) and G/T mutated variants (black) of model parallel quadruplex measured in 100K buffer at 23 °C.

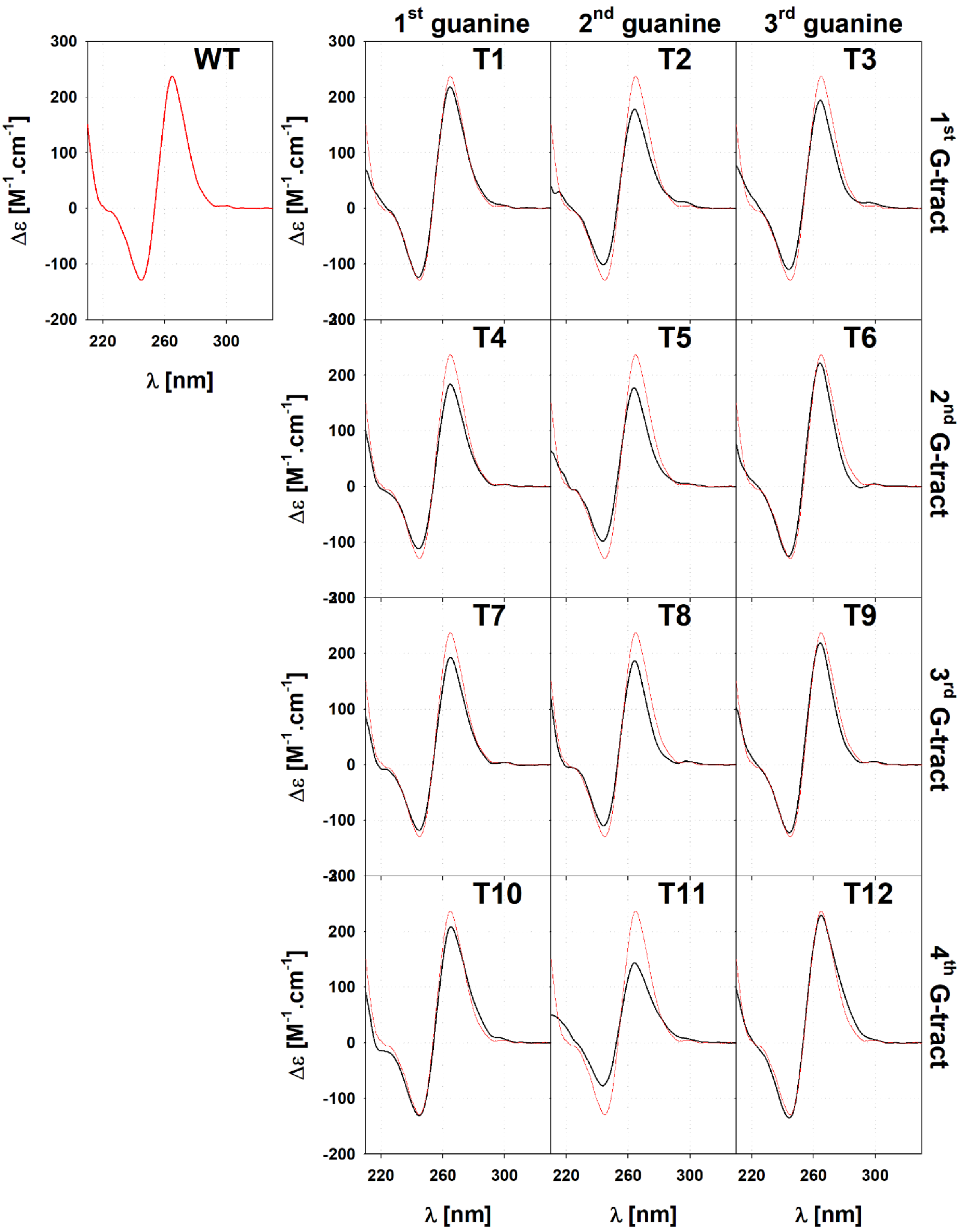

**Figure S7.** Averaged UV melting curves from three experiments of G/T mutated variants measured in 100K buffer and expressed as folded fraction (0-1 normalized curves) of G4 during renaturation (black) and denaturation (red). WT is not shown due to extreme thermal stability.

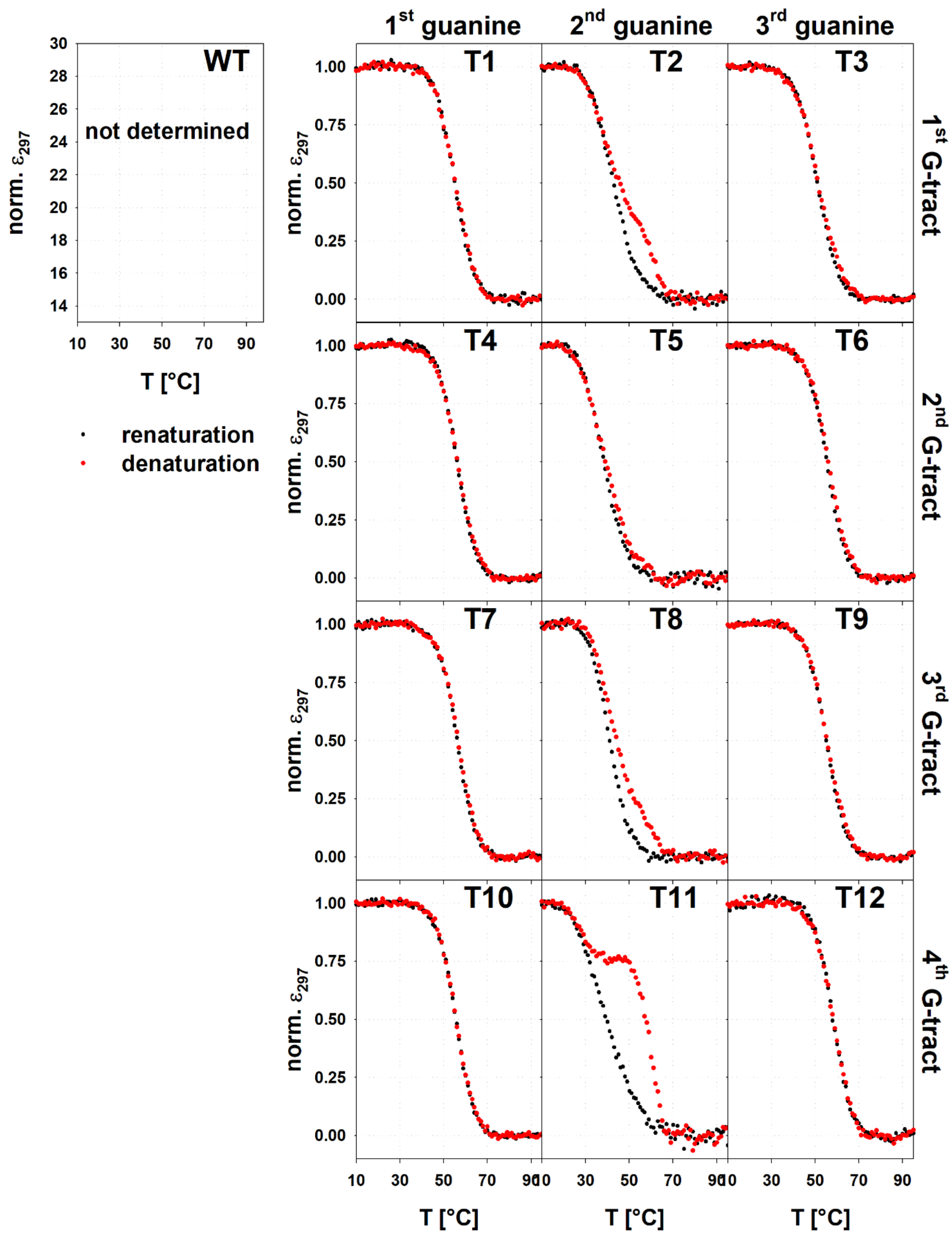

**Figure S8.** Native PAGE performed in 100K buffer at 20 °C (upper panel) or at 2 °C (bottom panel). Oligonucleotides were annealed in 100K buffer over 3 h from 90 °C to 20 °C or 2 °C.

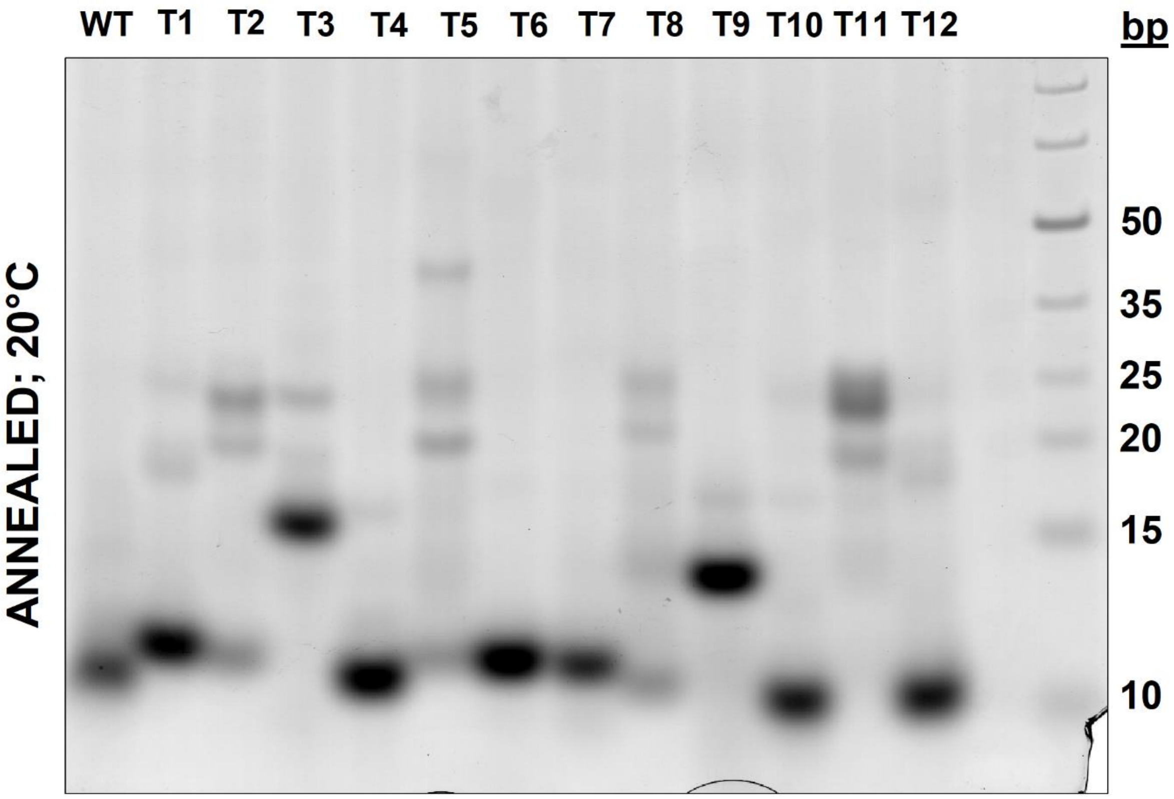


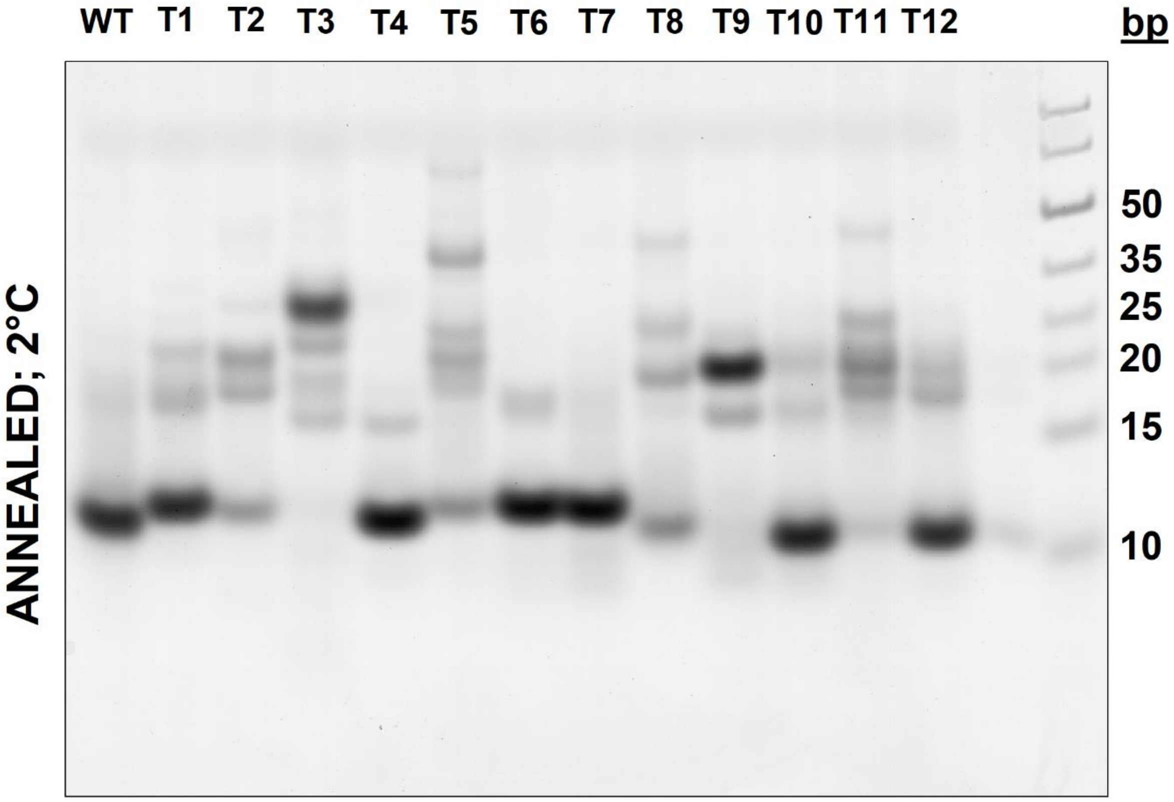

**Figure S9.** Left panels: Δε at 265 nm measured each millisecond for 5 s total after mixing sample with 2 mM $K^+$ (blue), 20 mM $K^+$ (red) or 200 mM $K^+$ (green) using a stopped-flow accessory. Black lines represent a three-parameter exponential fits ("rise-to-maximum") of the experimental points. Right panels: CD spectra of respective samples measured right after particular stopped-flow experiments. Black solid spectrum represents average of three independent measurements of sample prepared in 100K buffer by standard procedure.

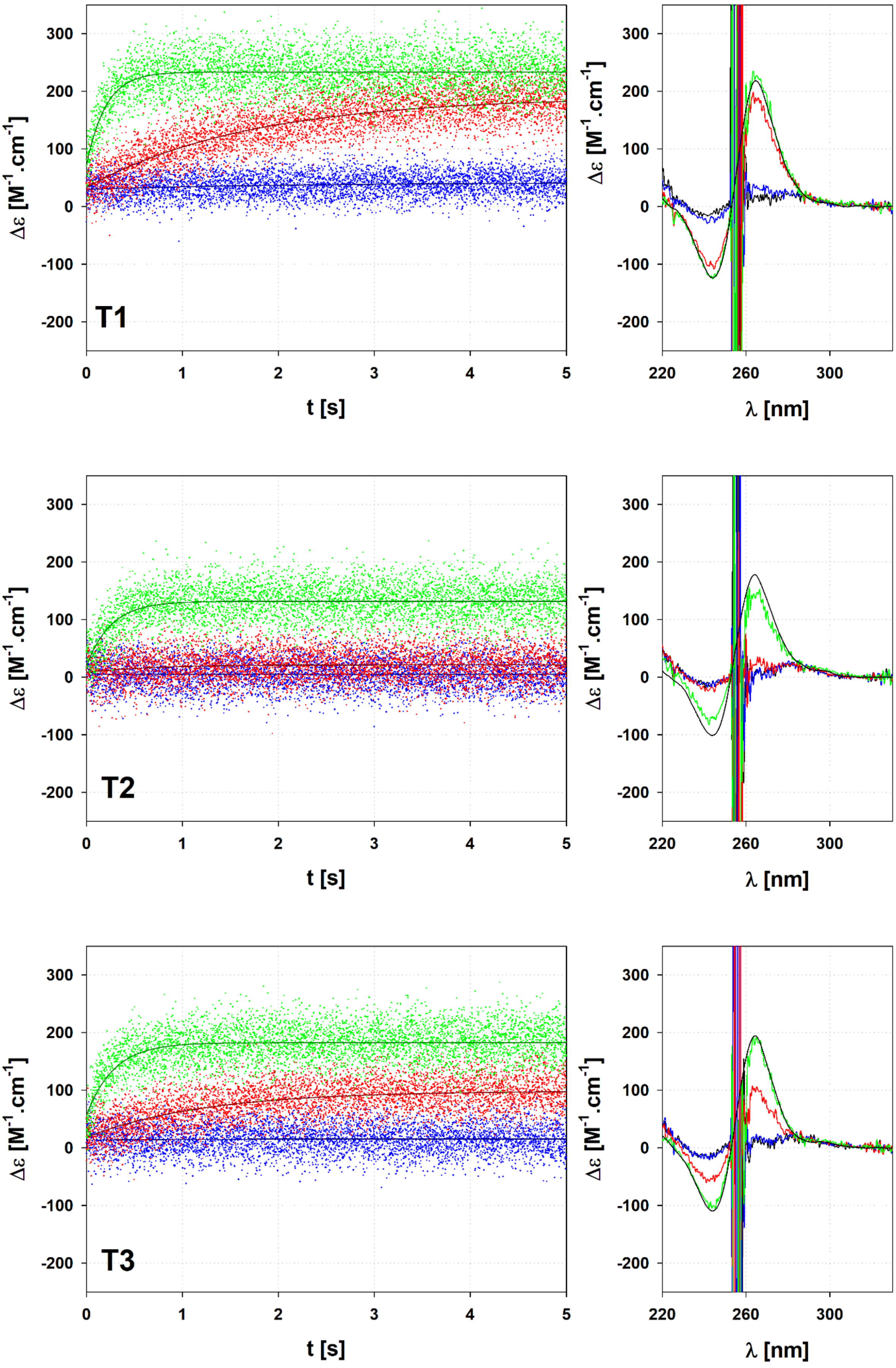
T1
T2
T3
Δε [M-1.cm-1]
t [s]
λ [nm]
300
200
100
0
-100
-200
0
1
2
3
4
5
220
260
300

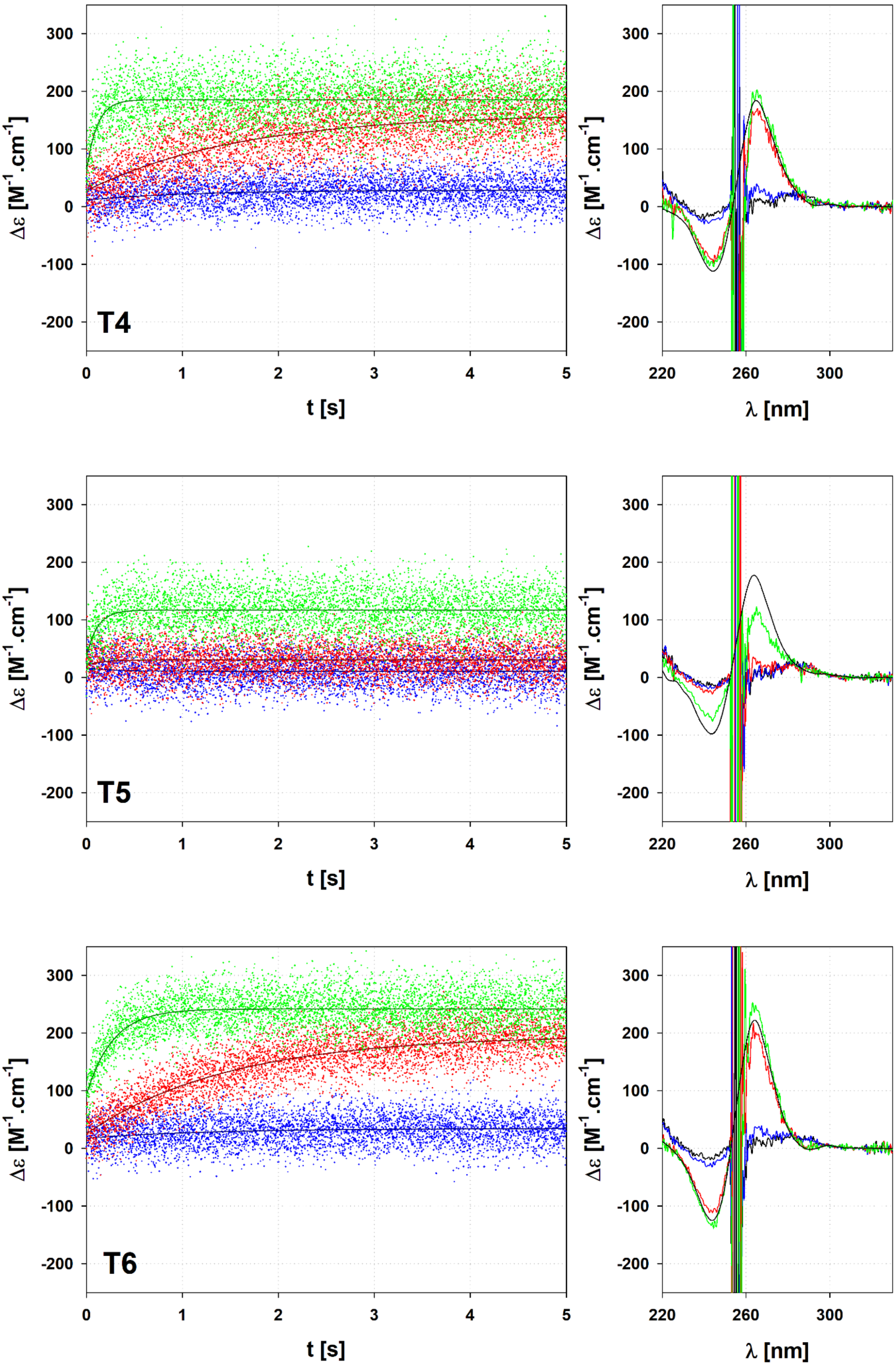

T4
T5
T6
Δε [M-1.cm-1]
t [s]
λ [nm]

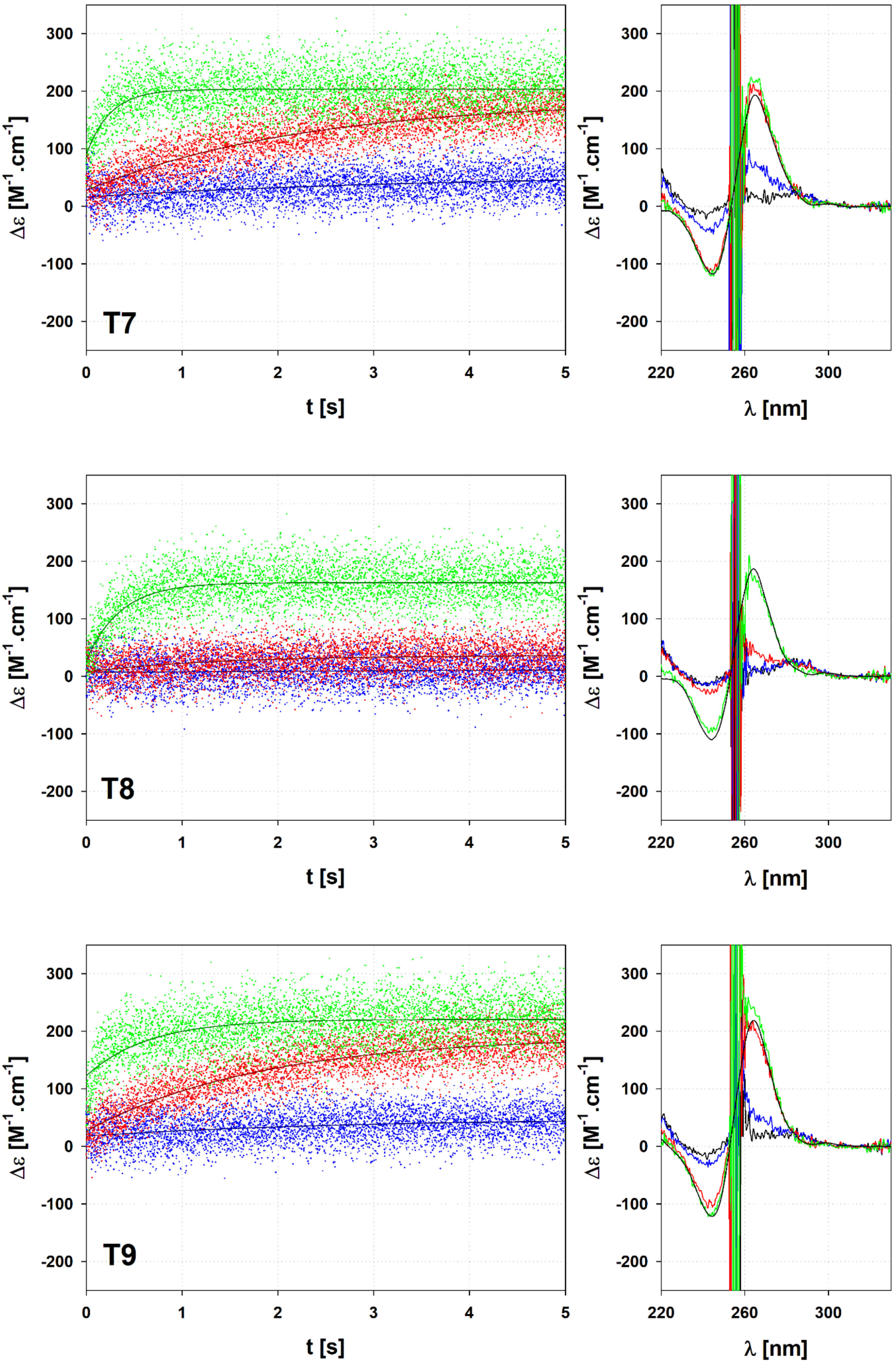
T7
T8
T9
Δε [M$^{-1}$.cm$^{-1}$]
300
200
100
0
-100
-200
0
1
2
3
4
5
t [s]
220
260
300
λ [nm]

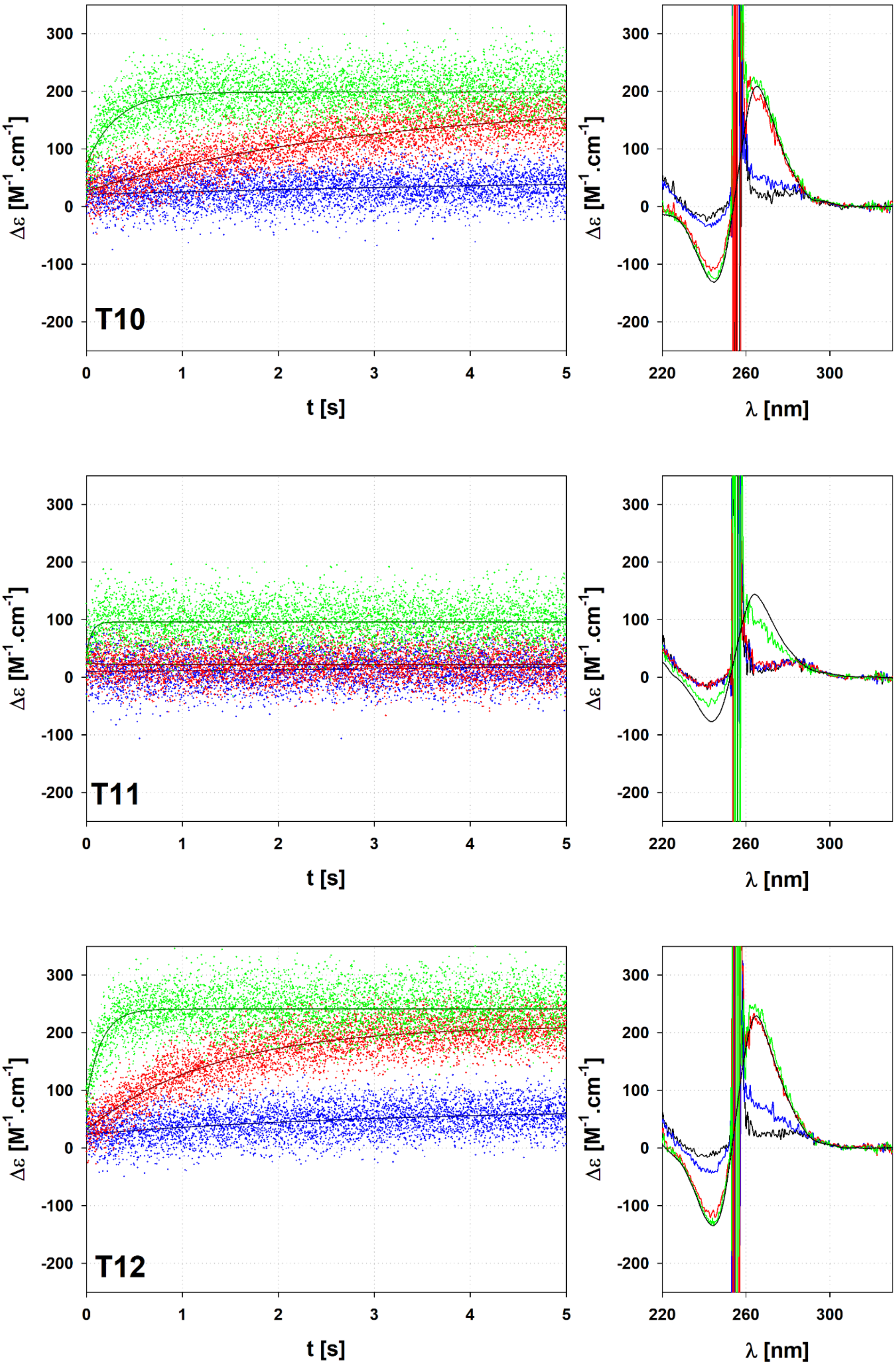
T10
T11
T12
Δε [M-1.cm-1]
300
200
100
0
-100
-200
0
1
2
3
4
5
t [s]
220
260
300
λ [nm]

**Figure S10.** The relative portion of G4 folded calculated from absorbance for all G/T mutants in 10K (top) and 100K (bottom) buffer at various times after mixing. The values are based on $\varepsilon_{265}$, normalized to value observed by stopped-flow experiment mixing DNA with 1NF buffer (0) and to value measured after annealing in 100K buffer (100).

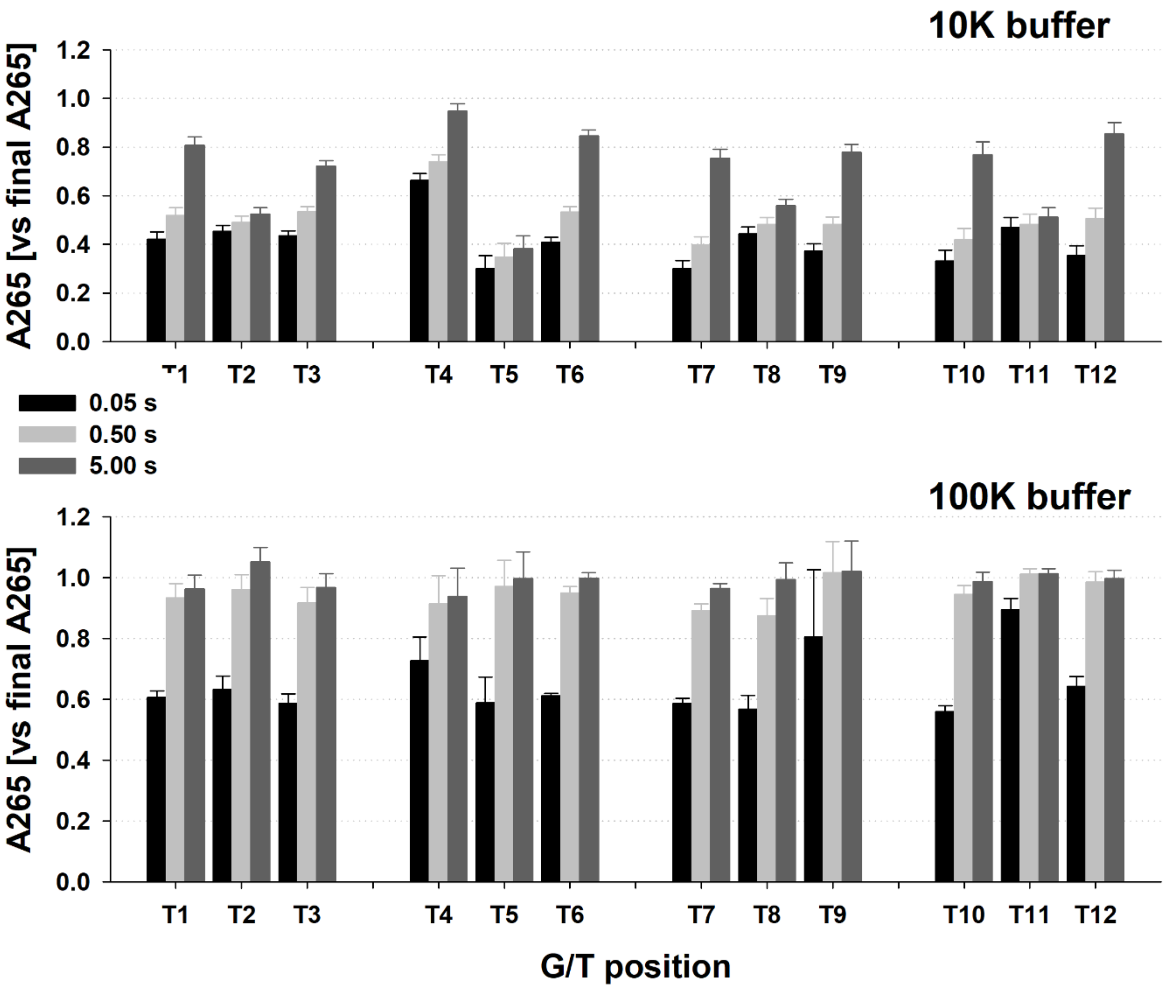

**Figure S11.** (**A**) The CD264 value (molar DNA strand circular dichroism (Δε) measured at 264 nm) of G to T substituted variants measured in 100K in absence (black) or presence (gray) of two equivalents of NMM. (**B**) Difference in $T_m$ values calculated from samples with and without two equivalents of NMM from renaturation (black) or denaturation (gray) profile measured in 100K buffer. $T_m$ of WT is not shown due to extreme thermal stability.

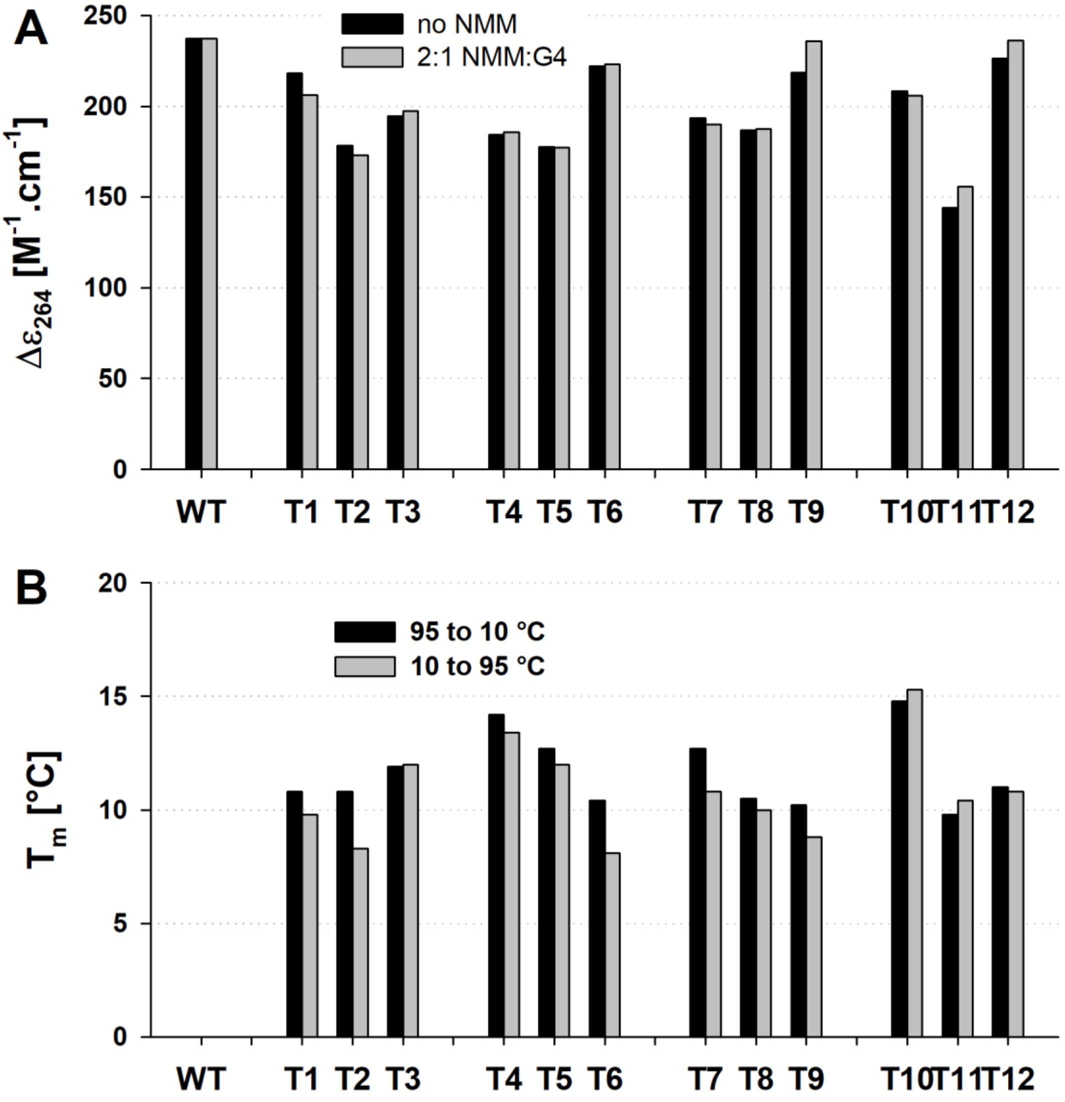

**Figure S12.** UV melting curves of G/T mutated variants measured in 100K buffer at various concentration and expressed as folded fraction (0-1 normalized curves) of G4 during renaturation (black) and denaturation (red). WT is not shown due to extreme thermal stability.

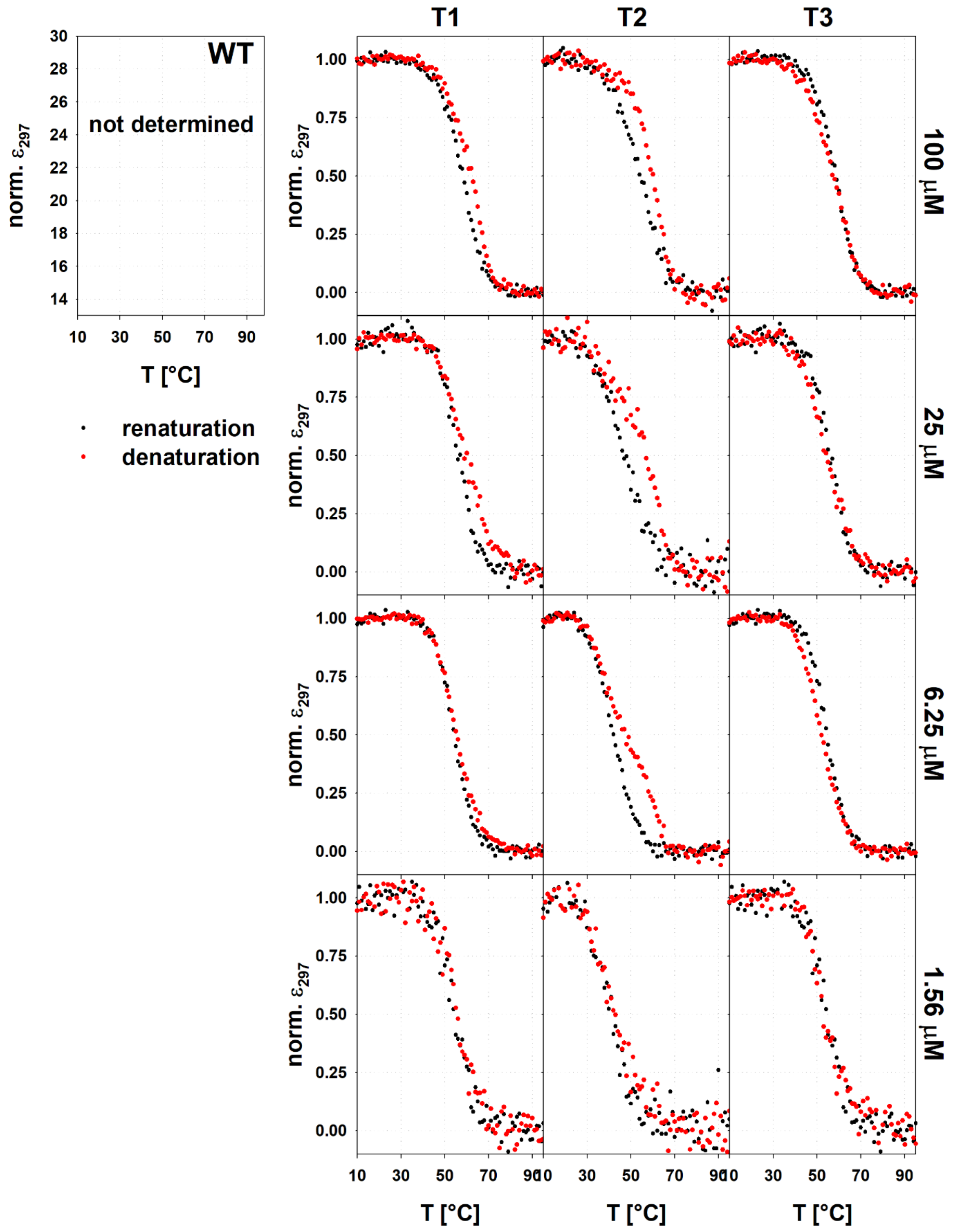

**Figure S13.** CD spectra of aWT (red) and G/T mutated variants (black) of model hybrid/antiparallel quadruplex measured in 100K buffer at 23 °C.

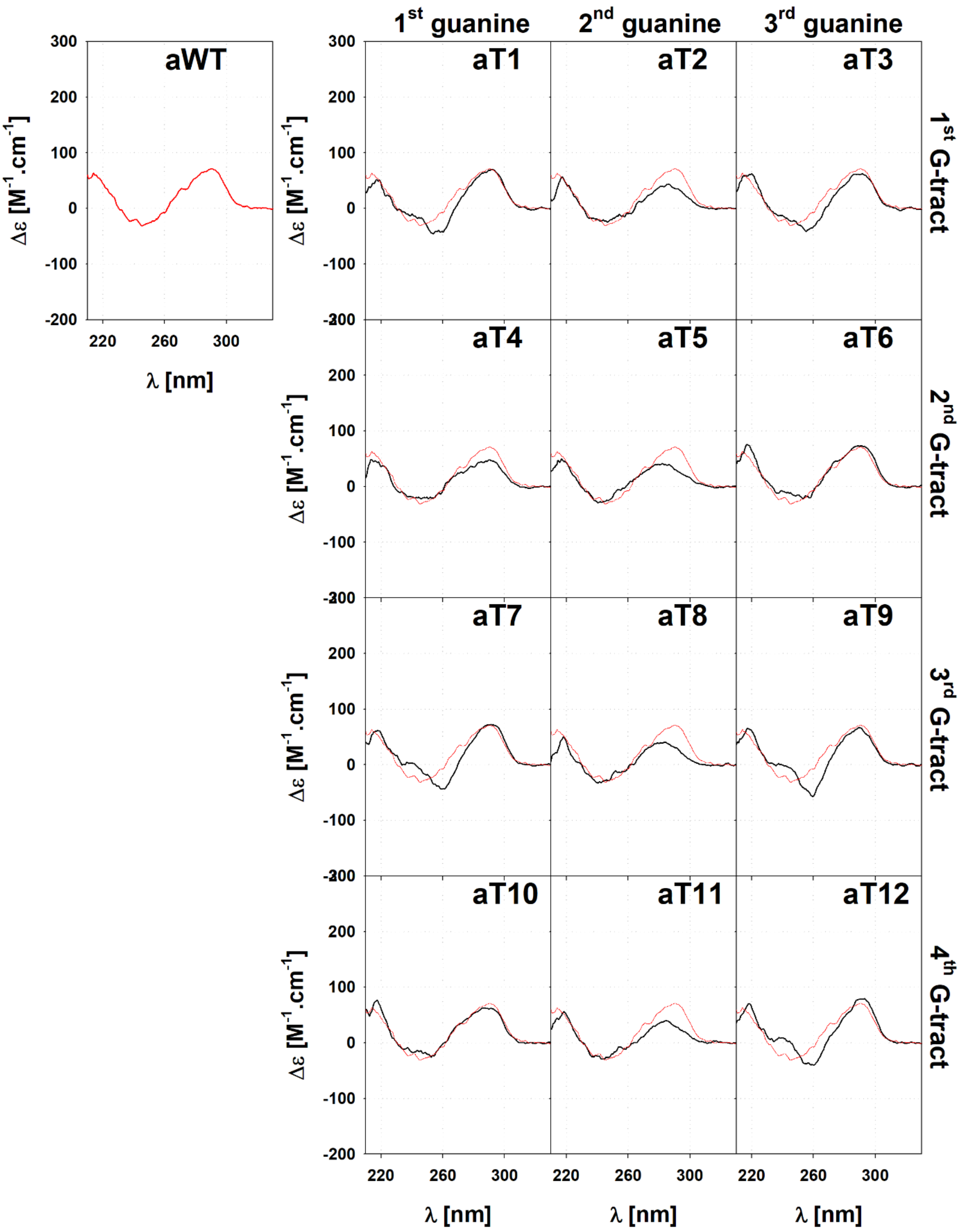

**Figure S14.** UV melting curves of aWT and G/T mutated variants measured in 100K buffer and expressed as folded fraction (0-1 normalized curves) of G4 during renaturation (black) and denaturation (red).

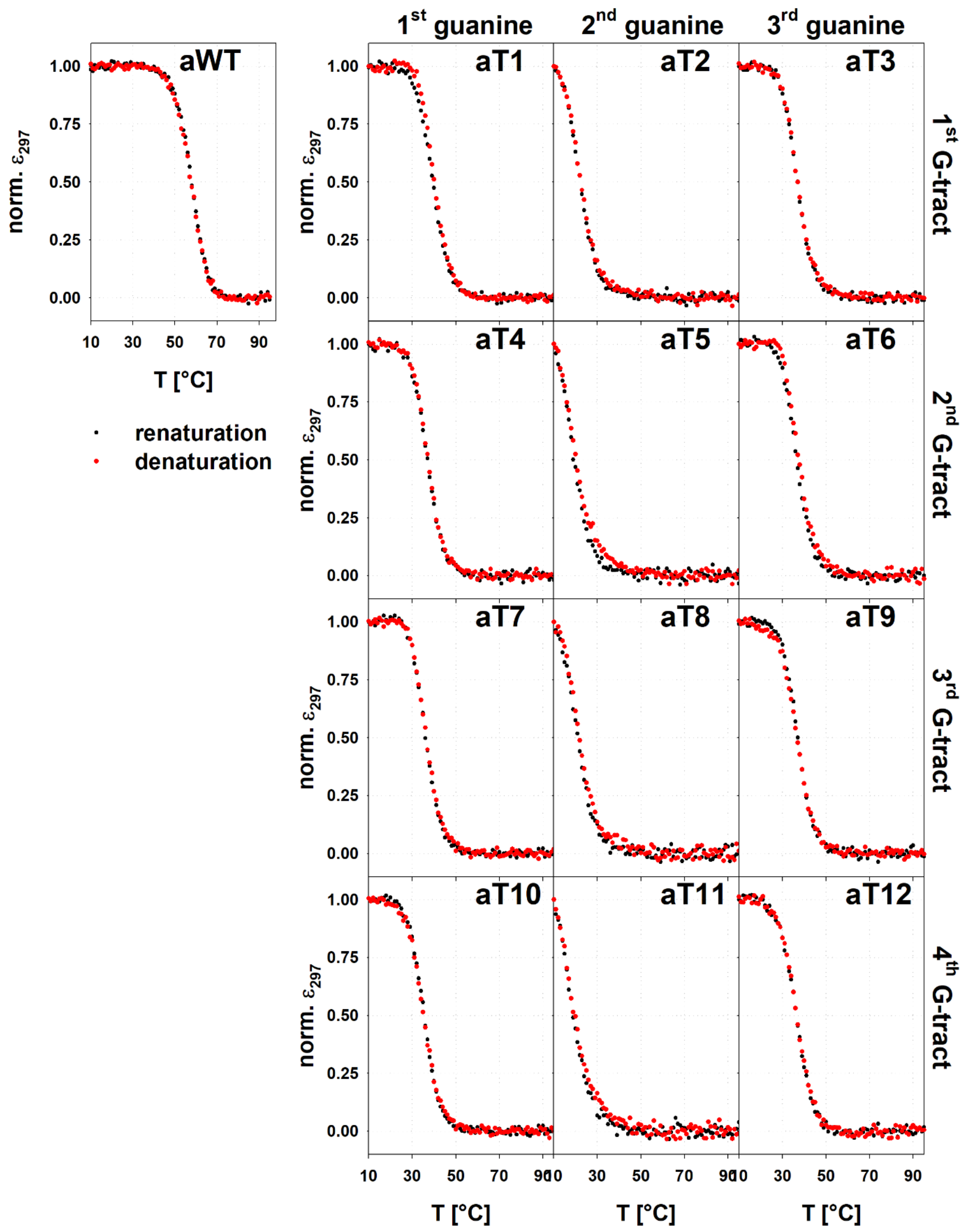

Supplement: Supplementary file 1 [file ijms-21-06123-s001.pdf]
